# ADAM12 is a costimulatory molecule that determines Th1 cell fate and mediates tissue inflammation

**DOI:** 10.1038/s41423-020-0486-8

**Published:** 2020-06-22

**Authors:** Yawei Liu, Robert Bockermann, Mahdieh Hadi, Iman Safari, Belinda Carrion, Marie Kveiborg, Shohreh Issazadeh-Navikas

**Affiliations:** 1 Neuroinflammation Unit, Biotech Research & Innovation Centre (BRIC), Faculty of Health and Medical Sciences, University of Copenhagen, Ole Maaløes Vej 5, DK-2200 Copenhagen N, Denmark; 2 BRIC, Faculty of Health and Medical Sciences, University of Copenhagen, Ole Maaløes Vej 5, DK-2200 Copenhagen N, Denmark

**Keywords:** T cells, T-bet, Th17, EAE, DTH, GM-CSF, Inflammation, Immunology

## Abstract

A disintegrin and metalloproteinase (ADAM)12 was previously found to be expressed in T cells in the inflamed brain. However, the function of ADAM12 in T-cell responses in general and in tissue inflammation has not been examined. Here, we studied the role of ADAM12 in T-cell responses, fate determination on activation, and its functions in T cells to mediate tissue inflammation. We identified ADAM12 as a costimulatory molecule that is expressed on naive T cells and downregulated on stimulation. ADAM12 mimics CD28 costimulatory signaling to activate and induce the proliferation of T-helper 1 (Th1) cells. Monoclonal ADAM12 Fab antibodies trigger T-cell activation by amplifying TCR signaling to stimulate T-bet-mediated IFNγ production. Lack of genomic *ADAM12* and its knockdown in T cells diminished T-bet and IFNγ production in Th1 cells, whereas other T cells, including Th17 cells, were unaffected. ADAM12 had similar functions in vivo on myelin antigen (MOG_35–55_)-induced T-cell activation. We found that genetic loss of *ADAM12* profoundly alleviated Th1-mediated neuroinflammation and thus disease severity in experimental autoimmune encephalomyelitis, a model of multiple sclerosis. Transcriptomic profiling of MOG_35–55_-specific ADAM12^−/−^ T cells revealed differentially expressed genes that are important for T-cell activation, proliferation, and costimulatory signaling and Th1 pathogenicity, consistent with their inability to cause T-cell-mediated skin inflammation in a model of adoptive delayed-type hypersensitivity. We conclude that ADAM12 is a T-cell costimulatory molecule that contributes to the pathogenesis of tissue inflammation and a potential target for the treatment of Th1-mediated diseases.

## Introduction

T lymphocytes are central in exerting the proper immune responses against microbial infections and during cancer progression. Thus, full activation of T cells via antigen recognition and T-cell receptor (TCR) and costimulatory signaling is essential to mount suitable immune responses.

Several transmembrane cluster of differentiation (CD) molecules, such as CD4, CD8, CD3, and CD7, are required for or contribute to TCR signaling. Further, a prerequisite of effective T-cell activation is the capacity of T cells to establish close contact or synapses with antigen-presenting cells (APCs).^1^ To this end, ligands or receptors that are commonly referred to as costimulatory molecules on T cells, such as CD28, bind to CD80 or CD86 (B7.1/2) molecules on APCs and stimulate additional intracellular signaling, leading to complete T-cell activation.^1^ Whereas certain costimulatory interactions, such as CD28-B7s and CD40-CD40L, contribute to T-cell activation,^2^ others, such as CTLA-4-B7s and PD-1-PD-L1, result in negative signaling that terminates T-cell activation.^3^

Many adhesion molecules and their receptors potentially have similar functions—although they might contribute to synapse formation and thus T-cell activation,^4^ they could synergize with chemokines and their receptors to elicit the migration of activated T cells to tissues to fight infections and cancer and mediate tissue healing. Activated T cells, in turn, are programmed to perform specialized effector functions, depending on the type of infectious/tumor antigen and the presence of costimulatory signaling.^5^

The induction of inappropriate or undesired T-cell effector functions, a lack of proper effector functions, and chronic activation of effector T cells can cause pathologies. Activation of self-antigen-reactive T cells with various effector functions, such as Th1, Th2, and Th17, are central for the induction and maintenance of many autoimmune diseases that afflict millions of children and adults worldwide.^6^ Thus, understanding how T cells are properly activated and the molecular mechanisms of their specialized functions and by which they home to specific inflammatory/infected or cancer-stricken tissues is essential for designing effective therapies with minimal side effects.

Leukocyte infiltration of the central nervous system (CNS) is a hallmark of autoimmune disease, multiple sclerosis (MS), and the MS model experimental autoimmune encephalomyelitis (EAE). To infiltrate the CNS parenchyma, leukocytes require proper activation to gain the capacity to migrate across the blood–brain barrier (BBB), which, under normal circumstances, restricts the entrance of cells and large molecules into the CNS. Disruption of the integrity of the BBB accompanies the extensive cellular infiltration and pathology that are seen in MS and EAE and is assumed to be a prerequisite for disease.^7^

Given their central nature in EAE, much MS/EAE research has focused on CD4^+^ T cells.^8^ Myelin-specific CD4^+^ Th1 cells are sufficient to induce EAE, which is evoked on adoptive transfer of such cells into naive recipient mice.^9^ T-helper 17 (Th17) cells have also been demonstrated to be important for the development of EAE.^10^

By RNA profiling, among 22 matrix metalloproteinases (MMPs) and 7 ADAM (a disintegrin and metalloproteinase) isoforms in EAE-induced mice, ADAM12 was found to be expressed almost exclusively by T cells.^11^ ADAM12 is a member of the ADAM family of MPs, which act as both proteases and signaling mediators, regulating cell fate.^12^ In EAE and MS, T cells and other leukocytes use MMPs to penetrate the BBB,^13^ increase cellular infiltration, and cause CNS pathology. However, there are limited data on the function of ADAM12 in T cells.

Existing research on ADAM12 in T cells has addressed the involvement and regulation of TGF-β signaling.^14^ TGF-β is a powerful inducer of FoxP3^+^ regulatory T cells (FoxP3^+^T_regs_)^15^ and has important functions in the differentiation of Th17 cells.^16^ Accordingly, the function of TGF-β signaling and ADAM12 in human FoxP3^+^ T_regs_ and Th17 has been studied in vitro. This report showed that ADAM12 mRNA is highly expressed by human FoxP3^+^T_regs_ and Th17 cells and that shRNA-mediated knockdown of ADAM12 increases IL-17 production by these cells.^14^ However, the precise function of ADAM12 in effector T cells and, consequently, in regulating inflammation CNS in vivo has not been examined.

In this study, we determined whether ADAM12 is expressed on the T-cell surface and examined its function in T-cell activation, proliferation, and inflammatory function. Unexpectedly, we did not find a major function for ADAM12 with regard to the capacity of T cells to migrate in the skin and parenchyma of CNS tissues, but instead discovered a previously unknown function of ADAM12 as a T-cell costimulatory molecule that amplifies TCR signaling and thus mediates the activation and proliferation of Th1 cells, enabling them to cause tissue inflammation.

## Results

### ADAM12 on T cells is downregulated on activation

ADAM12 has only been reported at the mRNA level in T cells.^11,14^ To determine the function of ADAM12 in T cells, *Adam12* mRNA was measured in spleens and CD4^+^ T cells. Notably, compared with *Cd28*, *Adam12* mRNA was expressed at lower levels in spleens and CD4^+^ T cells, near *Pdl1* levels (Supplementary Fig. 1a–c). However, *Adam12* mRNA was several-fold higher in purified CD4^+^ T cells versus splenocytes (Supplementary Fig. 1d).

**Fig. 1:**
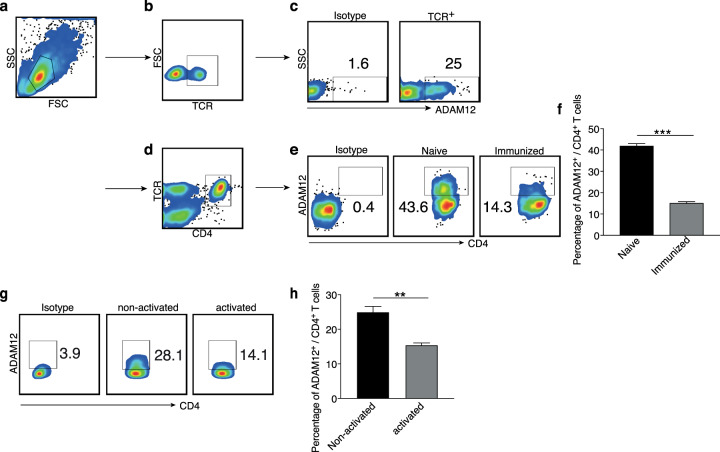
The expression of ADAM12 on T cells is downregulated on immunization. Spleens were taken from naive and MOG_35–55_-immunized C57BL/6 mice. Single-cell suspensions were stained with FACS antibodies. **a**, **b** FACS dot plots show the gating strategy; cells were gated on TCR^+^ cells. **c** Representative FACS dot plots show percentages of ADAM12 expression on TCR^+^ T cells. **d** FACS dot plots show T cells gated on TCR^+^ and CD4^+^. **e** Expression of ADAM12 in CD4^+^ T cells under naive and immunization conditions. **f** Percentage of ADAM12 expression in CD4^+^ T cells. **g** Expression of ADAM12 in CD4^+^ T cells and splenocytes treated or not with MOG_35–55_ (50 μg/ml) for 2 days. **h** Percentage of ADAM12 expression in CD4^+^ T cells after in vitro culture. Data are mean ± SEM from three independent experiments. **P < 0.01, ***P < 0.001 by Student’s *t* test

Next, we assessed whether ADAM12 could be detected at the protein level on T cells, examining spleens from naive and myelin oligodendrocyte glycoprotein (MOG)_35–55_-immunized mice by flow cytometry. As shown in gated T cells (TCR^+^ or CD4^+^), ADAM12 was expressed on the surface of T cells (Fig. 1a–c). Notably, ADAM12 expression was lower in immunized compared with naive mice (Fig. 1d-f). Further, in vitro-activated T cells also downregulated ADAM12 versus nonactivated T cells (Fig. 1g, h). This result indicates that ADAM12 is significantly influenced by T-cell activation and that ADAM12 might be required for activation. In support of these findings, clonally expanded T cells have been reported to undergo a program of persistent TCR downregulation.^17^

### ADAM12 acts as a costimulatory molecule, contributing to T-cell activation by amplifying TCR signaling

**Fig. 2:**
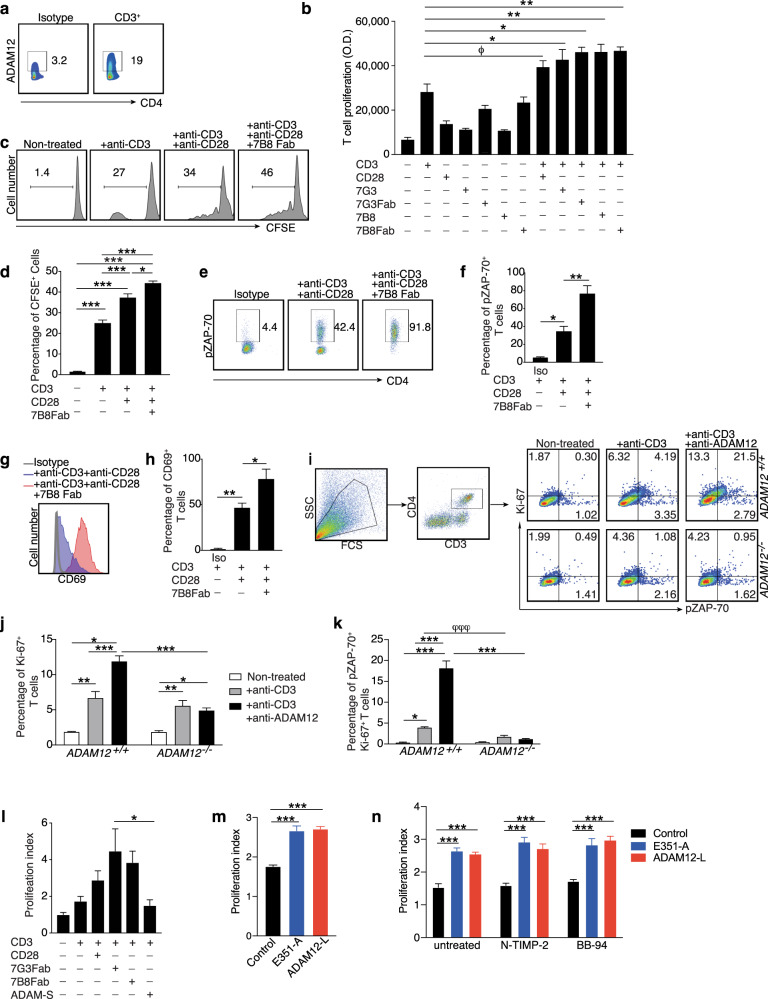
ADAM12 acts as a costimulatory molecule, contributing to T-cell activation by amplifying TCR signaling. Human CD4^+^ T cells were purified from healthy donors and seeded on plate-bound anti-CD3 (5 μg/ml) and soluble anti-CD28 (2 μg/ml) for 6 days, and anti-ADAM12 7G3 (10 μg/ml, full antibody or Fab), 7B8 (10 μg/ml, full antibody or Fab), and ADAM12-S (5 ng/ml) were added to the cultures separately. Cells were pulsed with BrdU for another 6 h. **a** Representative FACS dot plots of percentages of ADAM12 expression on untreated CD3^+^CD4^+^ T cells. **b** DNA synthesis was measured on an ELISA reader; proliferation assay results shown as O.D. values. Data are mean ± SEM of five independent experiments. *N* = 9; one-way ANOVA with Bonferroni’s multiple comparisons test was used, *P < 0.05, **P < 0.01. Student’s *t* test was used to show significant differences between anti-CD3 and anti-CD3 + anti-CD28, ^ϕ^*P* < 0.05. Human CD4^+^ T cells were purified from healthy donors. CD4^+^ T cells were labeled with CFSE prior to being seeded on plate-bound anti-CD3 (5 μg/ml), and soluble anti-CD28 (1 μg/ml) and 7B8 (10 μg/ml) were added to the cultures for 4–5 days as indicated. **c** Representative FACS dot plots of CFSE gating. **d** Quantification of percentage of CFSE^+^ cells. **e** Representative FACS dot plots of pZAP-70 staining as indicated. **f** Quantification of percentage of pZAP-70^+^ CD4^+^ T cells. **g** Representative FACS dot plot of CD69 staining as indicated. **h** Quantification of percentage of CD69^+^CD4^+^ T cells. Graphs in **d**, **f**, and **h** are mean ± SEM from three independent experiments. *N* = 3–4 per group. *P < 0.05, **P < 0.01, ***P < 0.001 by one-way ANOVA with post-Tukey’s test. *ADAM12*^+/+^ and *ADAM12*^*−/−*^ spleens were taken from MOG_35–55_-induced EAE mice. Single-cell suspensions were treated with plate-bound anti-CD3 (5 μg/ml) and anti-ADAM12 (10 μg/ml) for 3 days prior to being stained with FACS antibodies. **i** FACS dot plots of the gating strategy; cells were gated on CD3^+^CD4^+^ T cells. Representative FACS dot plots show percentages of Ki-67 and pZAP-70 expression on T cells under various conditions. **j** Percentage of Ki-67^+^ and **k** pZAP-70^+^Ki-67^+^ cells. Data are mean ± SEM from three independent experiments. *N* = 3 was used. *P < 0.05, **P < 0.01, ***P < 0.001 by two-way ANOVA and post-Tukey’s multiple comparisons test. Student’s *t* test was used to analyze CD3 treatment effects between *ADAM12*^*+/+*^ and *ADAM12*^*–/–*^ T cells. ^φφφ^*P* < 0.001. **l** DNA synthesis was measured on an ELISA reader; proliferation assay results are shown as O.D. values. Data are mean ± SEM from four independent experiments. *N* = 9. *P < 0.05 by one-way ANOVA with Bonferroni’s multiple comparisons test. Jurkat cells were transfected with wild-type (ADAM-L) and catalytically inactive (E351-A) full-length human ADAM12-L constructs. After 48 h, Jurkat T cells were stimulated overnight with ImmunoCult™ Human CD3/CD28 T-Cell Activator. **m** BrdU cell proliferation assay; relative values calculated as OD value divided by OD value in non-treated cells. Data are mean ± SEM from two independent experiments. *N* = 6. ***P < 0.001 by one-way ANOVA with post-Tukey’s test. **n** N-TIMP-2 (100 nM) and BB-94 (20 nM) were added to the cultures overnight prior to the cell proliferation assay. Data are mean ± SEM from two independent experiments. *N* = 6. ***P < 0.001 by two-way ANOVA and post-Tukey’s multiple comparisons test

To determine the impact of ADAM12 on T-cell activation and proliferation, we exploited monoclonal antibodies against ADAM12 (7B8 and 7G3) that bind its extracellular domain.^18^ Human CD4^+^ T cells were purified, and ADAM12 protein was detected on T cells (Fig. 2a). CD4^+^ T cells were labeled with BrdU or carboxyfluorescein diacetate succinimidyl ester (CFSE) to analyze cell proliferation.

Notably, the combination of anti-ADAM12 and anti-CD3 induced T-cell proliferation to the same degree as that of anti-CD3 and anti-CD28, which triggers both required T-cell signals for full activation and thereby proliferation^1^—i.e., the TCR and costimulatory signals (Fig. 2b). Whereas the ADAM12 antibodies (the entire IgG or Fab fragment) fully replaced and mimicked the function of the costimulatory signal (anti-CD28), anti-ADAM12 alone (without anti-CD3) failed to induce T-cell proliferation (Fig. 2b). We found no significant difference in effects between the whole antibody or Fab fragment, indicating that no cross-linking of the receptor is required.

To confirm the function of ADAM12 as a costimulatory molecule, we assessed the expansion of CFSE-labeled T cells. Again, we found that combining the anti-ADAM12 Fab fragment with the classical T-cell signaling factors (anti-CD3 and anti-CD28) induced significant proliferation of T cells (Fig. 2c, d).

To further examine T-cell signaling on treatment with anti-ADAM12 Fab, ZAP-70 was measured as a reflection of TCR signaling. ZAP-70 is a cytoplasmic protein tyrosine kinase that is critical in the initiation of a T-cell response.^19,20^ The kinase domain of ZAP-70 can be phosphorylated and function as a scaffold to recruit other signaling molecules, leading to T-cell activation, proliferation, and differentiation.^20^ Here, phosphorylated ZAP-70 was significantly upregulated on addition of anti-ADAM12 Fab (Fig. 2e, f), indicating that this antibody facilitates costimulation and thus further increases TCR signaling. Also, costimulation with anti-ADAM12 Fab upmodulated the T-cell activation marker CD69 (Fig. 2g, h).

To exclude the possibility that the ADAM12 antibodies crossreact with other molecules, we purified murine CD4^+^ T cells from wild-type (*ADAM12*^*+/+*^) and *ADAM12*^*−/−*^ mice and treated them with anti-CD3, alone or with anti-ADAM12 Fab. Indicating the specificity of the induced signal, the lack of genomic *ADAM12* in T cells prevented the generation of a costimulatory signal on antibody treatment; thus, the T cells were not proliferating (Ki-67^+^) or transducing intracellular signals, as measured by pZAP-70 positivity in Ki-67^+^ T cells (Fig. 2i–k).

Human ADAM12 has two isoforms: the prototypical transmembrane ADAM12-L and a short, secreted version, ADAM12-S, which lacks the cytoplasmic tail and transmembrane domain.^21^ To examine whether ADAM12-S is sufficient for triggering a costimulatory signal, T cells were treated with purified recombinant ADAM12-S. However, this isoform did not have any impact (Fig. 2l), indicating that ADAM12-L is required.

Next, to determine whether the proteolytic activity of ADAM12 is involved in its induction of T-cell costimulatory signals, we overexpressed full-length wild-type transmembrane ADAM12 (ADAM12-L) or catalytically inactive mutant ADAM12 (ADAM12 E351-A) in Jurkat cells. To verify the inactivity of ADAM12, a cell-based epidermal growth factor (EGF) shedding assay was performed. As reported,^22^ no proteolytic activity of the ADAM12 E351-A mutant was observed, whereas wild-type ADAM12 shed EGF reporter efficiently into the media (Supplementary Fig. 1e). Notably, both wild-type ADAM12 and ADAM12 E351-A induced Jurkat cell proliferation (Fig. 2m), indicating that this effect is independent of its proteolytic activity. Confirming this finding, the metalloprotease inhibitors N-terminal domain of the tissue inhibitor of metalloproteinase-2 (N-TIMP-2) and batimastat (BB-94), both of which inhibit the catalytic activity of ADAM12,^22^ did not impede ADAM12-induced proliferation of Jurkat cells (Fig. 2n).

Collectively, these observations demonstrate that ADAM12 is a costimulatory signaling molecule that mediates T-cell activation and proliferation by amplifying TCR signaling independently of its proteolytic activity.

### ADAM12 is essential for T-bet expression and IFNγ production in T cells

**Fig. 3:**
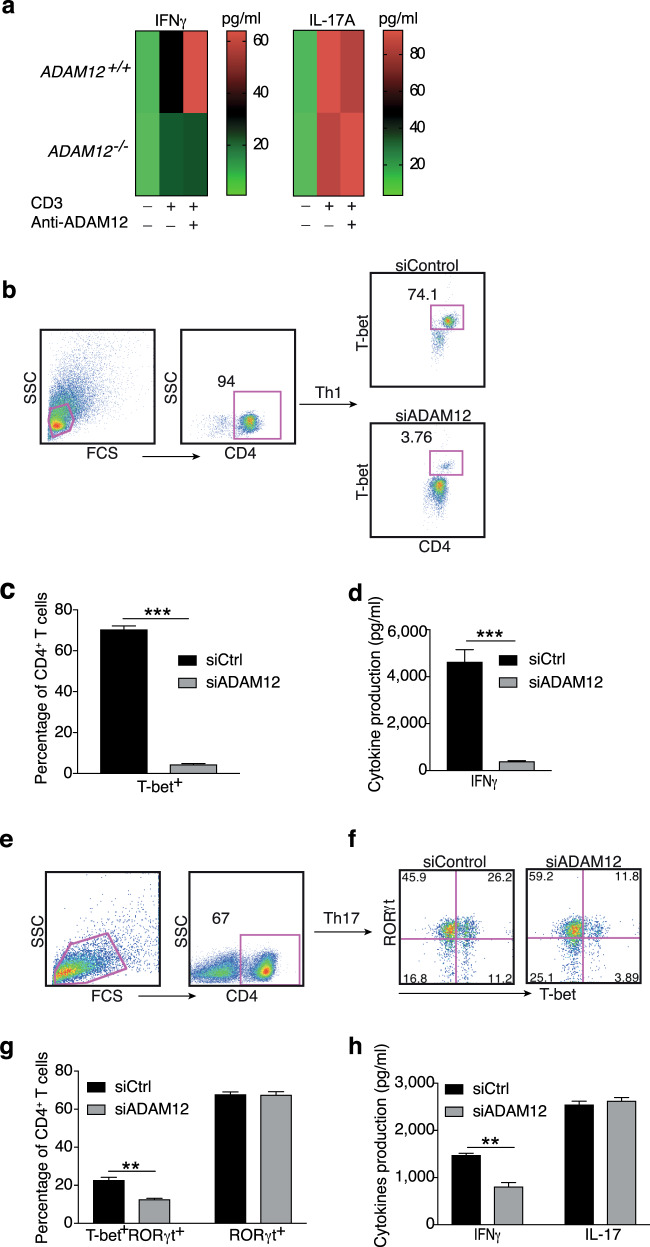
ADAM12 is essential for T-bet expression in T cells and IFNγ production. **a** *ADAM12*^*+/+*^ and *ADAM12*^*–/–*^ T cells were treated with plate-bound anti-CD3 (5 μg/ml) and anti-ADAM12 (10 μg/ml) for 3 days. Cell-culture supernatants were collected for ELISA. MOG_35–55_-specific T cells treated with MOG_35–55_ (50 μg/ml), IL-12 (20 ng/ml), anti-IL-4 (10 μg/ml), and siRNAs (siControl or siADAM12) for 72 h. **b** FACS dot plots of the gating strategy and T-bet staining in CD4^+^ T cells. **c** Quantification of FACS results from **b**. **d** IFNγ production in Th1 cell cultures by ELISA. Graphs in **c, d** are mean ± SEM from three independent experiments. ***P < 0.001 by Student’s *t* test. Th17 cells polarized in vitro. **e**, **f** Representative FACS plots of gating strategy and T-bet- and RORγt-stained CD4^+^ T cells. **g** Quantification of FACS results from a representative experiment—i.e., from two independent experiments. Graphs are mean ± SEM, *N* = 3. **h** IFNγ and IL-17 production in Th17 cell cultures, by ELISA from three independent experiments. **P < 0.01 by two-way ANOVA with post-Tukey’s multiple comparisons test

We examined the effector profile that is established by ADAM12 signaling. Among the panel of cytokines that was tested, only IFNγ production was defective in antigen-stimulated *ADAM12*^*−/−*^ T cells; no differences in IL-10, TGF-β1, IL-4, or IL-17 were seen (Fig. 3a; Supplementary Fig. 1f–i).

To verify that this finding was the direct result of ADAM12 costimulatory signaling in mature T cells and not a defect in T-cell maturity during development, we knocked down (KD) ADAM12 by siRNA in purified matured murine CD4^+^ T cells. We observed that ADAM12 in CD4^+^ T cells was efficiently knocked down (Supplementary Fig. 1j). Naive CD4^+^ T cells were then activated with anti-CD3/anti-CD28 and polarized under Th1 conditions for 3–4 days. Whereas CTLA-4, B7.1, PD-1, RORγt, Foxp3, and FoxA1 protein levels were unaffected by knockdown of ADAM12 (Supplementary Fig. 1k), T-bet expression was severely diminished in ADAM12 knockdown CD4^+^ cells under Th1-polarizing conditions (Fig. 3b, c). T-bet induces IFNγ by binding to the *Ifng* promoter.^23^ In accordance, IFNγ production was significantly reduced on ADAM12 knockdown (Fig. 3d). These results suggest that ADAM12 is essential for T-bet expression and IFNγ production and thus Th1 cell development.

T-bet is also expressed in RORγt^+^ Th17 cells, and only T-bet^+^RORγt^+^ double-positive cells are pathogenic T cells.^24^ Thus, we examined whether ADAM12 affects T-bet^+^RORγt^+^ double-positive cells in the Th17 population. As shown in Fig. 3e, f, knockdown of ADAM12 in Th17-polarized cells resulted in a significant reduction of T-bet^+^RORγt^+^ double-positive T cells, whereas RORγt^+^ single-positive T cells were not altered (Fig. 3g; Supplementary Fig. 1l, m). These results indicate that under Th1 and Th17 conditions, T-bet is the only molecular target of ADAM12 that is positively regulated—not RORγt. Also, ADAM12 knockdown-mediated suppression of T-bet significantly lowered IFNγ production but had no impact on IL-17 (Fig. 3h).

### ADAM12 regulates the severity of the neurological symptoms of EAE

In the T-cell-mediated CNS inflammatory disease EAE, a previous study has shown that ADAM12 mRNA levels are elevated by over threefold in CNS-infiltrating T cells in EAE versus naive mice,^11^ supporting that ADAM12 has important functions in inflammatory processes. In addition, we found that ADAM12 protein is expressed on T cells and has costimulatory function in T-cell proliferation, but that it is downregulated upon activation. Thus, we examined the function of ADAM12 in the regulation of CNS inflammation in EAE.

**Fig. 4:**
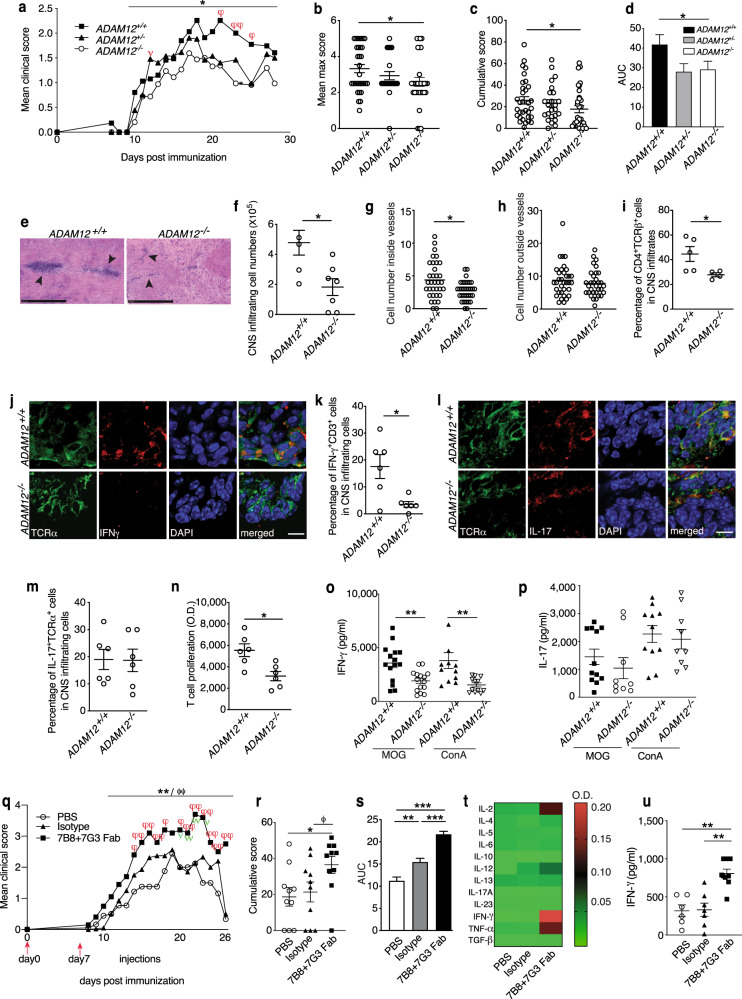
ADAM12 regulates the severity of neurological symptoms of EAE. Mean clinical EAE score in *ADAM12*^*+/+*^*, ADAM12*^*+/−*^, and *ADAM12*^*–/–*^ mice; *N* = 24–32 mice per group. *P < 0.05 by Kruskal–Wallis with post-Dunn’s multiple comparisons test between *ADAM12*^*–/–*^ and *ADAM12*^*+/+*^. Mann–Whitney test shows a significant difference on day 12 (*ADAM12*^*–/–*^ *vs ADAM12*^*+/–*^), ^ν^*P* < 0.05, and on days 21, 23, and 25, respectively (*ADAM12*^*–/–*^ vs *ADAM12*^*+/+*^), ^φ^*P* < 0.05, ^φφ^*P* < 0.01. **b** Mean max score. **c** Cumulative score and **d** area under the curve (AUC) were calculated separately in *ADAM12*^*+/+*^*, ADAM12*^*+/–*^, and *ADAM12*^*–/–*^ groups; data are mean ± SEM from two independent experiments. *N* = 24–33 mice per group. Kruskal–Wallis with post-Dunn’s multiple comparisons test shows a significant difference in mean max score between *ADAM12*^*–/–*^ and *ADAM12*^*+/+*^mice, *P < 0.05. Mann–Whitney test shows a significant difference in cumulative score and AUC between *ADAM12*^*–/–*^ and *ADAM12*^*+/+*^ mice, *P < 0.05. **e** Representative H&E images of spinal cords from mice with EAE; scale bars = 500 μm. **f** Cell counts after purification of CNS-infiltrating cells; data are mean ± SEM from three independent experiments. *N* = 6–7. *P < 0.05 by Student’s *t* test. Manual quantification of the number of CNS-infiltrating cells from histology slides **g** inside and **h** outside of vessels. *N* = 32–34 tissue sections per group. Data are mean. *P < 0.05 by Student’s *t* test. **i** FACS analysis of percentage of CD4^+^ T cells from purified CNS-infiltrating cells. Data are min to max from five independent experiments. *N* = 4–5. *P < 0.05 by Mann–Whitney test. **j** Immunofluorescence staining of IFNγ^+^TCRα^+^ T cells among CNS-infiltrating cells: TCRα (green), IFNγ (red), and DAPI (blue); scale bars = 10 μm. **k** Quantification of the number of IFN-γ^+^CD3^+^ among CNS-infiltrating cells in *ADAM12*^+/+^ and *ADAM12*^−/−^ EAE mice; data are mean ± SEM from three independent experiments. *N* = 6 mice per group. *P < 0.05 by Student’s *t* test. **l** Immunofluorescence staining of IL-17^+^TCRα^+^ T cells among CNS-infiltrating cells: TCRα^+^ (green), IL-17 (red), and DAPI (blue); scale bars = 10 μm. **m** Quantification of the number of IL-17^+^TCRα^+^T cells among CNS-infiltrating cells in *ADAM12*^+/+^ and *ADAM12*^−/−^ EAE mice; data are mean ± SEM from three independent different experiments. *N* = 6 mice per group. Spleens were taken from *ADAM12*^+/+^ and *ADAM12*^−/−^ EAE mice, single-cell suspensions were established and treated with MOG_35–55_ (/ml) for 48 hours, cells were pulsed with BrdU for another 6 h, and DNA synthesis was measured on an ELISA reader. **n** DNA synthesis (O.D. value) of T-cell proliferation in MOG_35–55_-treated splenocytes. Data are mean ± SEM from three independent experiments. *N* = 6. *P < 0.05 by Mann–Whitney test. Cell culture supernatants were collected, and **o** IFN-γ and **p** IL-17 production in splenocyte cell cultures was measured by ELISA. ConA (Concanavalin A, 5 μg/ml) was used as a positive control. Data are mean ± SEM from three independent experiments. *N* = 12–15. **P < 0.01 by Mann–Whitney test for comparison between *ADAM12*^+/+^ versus *ADAM12*^−/−^. **q** Mean clinical EAE score of C57BL/6 mice treated with MOG_35–55_ and anti-ADAM12 Fab (7B8 + 7G3). Purified monoclonal lgG was used as an isotype control. *N* = 10 mice per group. **P < 0.01 by Kruskal–Wallis test, showing significant difference between anti-ADAM12 Fab-treated and PBS groups. ^ϕϕ^*P* < 0.01 by Mann–Whitney test, showing significant difference between anti-ADAM12 Fab-treated and isotype control groups. Mann–Whitney test shows a significant difference on days 14, 15, 16, 17, 18, 20, 21, 22, 23, 24, and 25 (7B8 + 7G3 vs PBS)**:** ^φ^*P* < 0.05, ^φφ^*P* < 0.01 and on days 20, 21, 22, and 23 (7B8 + 7G3 vs isotype): ^ν^*P* < 0.05, ^νν^*P* < 0.01. **r** Cumulative score of three groups. Data are mean ± SEM; *N* = 10 mice per group. *P < 0.05 by Kruskal–Wallis test, showing significant difference between antibody-treated and PBS groups. ^ϕ^*P* < 0.05 by Mann–Whitney test, showing significant difference between antibody-treated and isotype control groups. **s** Area under curve (AUC) values were calculated separately. Graphs are mean ± SEM; *N* = 10 mice per group. **P < 0.01, ***P < 0.001 by one-way ANOVA with post-Tukey’s multiple comparison. **t** Spleens were taken from EAE mice, single-cell suspensions were established and treated with MOG_35–55_ (50 μg/ml) for 48 h, and cell culture supernatants were collected for ELISA. Th1, Th2, and Th17 cytokines were measured by ELISA in the supernatants, pooled for each group. Data are mean from ten splenocyte cultures. **u** IFNγ production by MOG_35–55_-treated splenocyte cultures. Graphs are mean ± SEM. *N* = 6–9. **P < 0.01 by Kruskal–Wallis test

To this end, we studied MOG_35–55_-induced EAE in *ADAM12*^*−/−*^ mice. We observed significantly lower disease severity in *ADAM12*^*−/−*^ mice compared with their *ADAM12*^*+/+*^ littermates, whereas the disease incidence did not differ (Fig. 4a–d and Table 1). Notably, the severity of disease was significantly lower in *ADAM12*^*−/−*^ mice in the acute phase of EAE and during the entire duration of EAE (area under the curve) compared with ADAM12 heterozygotes (*ADAM12*^*+/−*^) (Fig. 4a). Because there were no significant differences between *ADAM12*^*+/−*^ and wild-type (*ADAM12*^*+/+*^) mice (Fig. 4a–d and Table 1), *ADAM12*^*+/−*^ heterozygotes were not included in subsequent experiments.

**Table 1:** *ADAM12*^+/+^ mice develop more severe EAE than *ADAM12*^*−/−*^ animals

Group	Mean max score ± SD	Max score	Mean cumulative score	Incidence (number)
*ADAM12*^+/+^	3.33 ± 1.29	5	25.98 ± 19.36	100% (33/33)
*ADAM12*^+/−^	2.94 ± 1.13	5	23.08 ± 17	96% (23/24)
*ADAM12*^−/−^	2.59 ± 1.39*	5	17.84 ± 18.48*	90% (29/32)

Mean max score: *P < 0.05, *ADAM12*^*−/−*^ compared with *ADAM12*^+/+^ by Kruskal–Wallis test.

Mean cumulative score: calculated by summing each individual score registered during the follow-up period until day 28 post immunization, divided by the number of mice per group. *P < 0.05, *ADAM12*^*−/−*^ compared with *ADAM12*^+/+^ by Mann–Whitney test.

Our data implicate ADAM12 in the regulation of the clinical and neurological symptoms of EAE.

### Lack of ADAM12 does not affect the parenchymal migratory capacity of leukocytes

Pathogenic leukocyte infiltration of the CNS parenchyma is the key pathological feature of EAE and is central for its outcomes. Based on an earlier report,^11^ we hypothesized that ADAM12 regulates EAE by governing the CNS-infiltrating capacity of leukocytes. By hematoxylin and eosin (H&E) staining of CNS tissues in EAE mice, as expected, we observed significantly greater inflammation, consisting of more CNS-infiltrating cells in *ADAM12*^*+/+*^ versus *ADAM12*^*−/−*^ mice (Fig. 4e, f), supporting our observation of more severe disease in *ADAM12*^*+/+*^ compared with *ADAM12*^*−/−*^ mice (Fig. 4a–d).

To examine the effects of ADAM12 on the CNS parenchymal migratory capacity of leukocytes, we quantified the infiltrating cells inside and outside of blood vessels in the spinal cords of EAE mice (Supplementary Fig. 2a). We found significantly more cells in blood vessels and in close proximity to vessels in *ADAM12*^*+/+*^ versus *ADAM12*^*−/−*^ mice (Fig. 4f, g). However, the absence of ADAM12 did not lead to fewer infiltrating cells in the CNS parenchyma (outside of blood vessels; Fig. 4h). These data suggest that the lack of ADAM12 does not prevent infiltrating cells from migrating within the CNS parenchyma, challenging the hypothesis that ADAM12 is important for leukocytes to migrate within the parenchyma to induce neuroinflammation and disease.

Instead, our results indicate that ADAM12 mediates the activation of T cells in the periphery, but that its absence does not significantly impede their movement or function in the CNS parenchyma. In agreement, we did not find any significant differences in EAE-associated demyelination in the spinal cords of *ADAM12*^*+/+*^ and *ADAM12*^*+/−*^ mice, compared with *ADAM12*^*−/−*^ animals (Supplementary Fig. 2b), arguing against a major function for ADAM12 expression by immune cells and oligodendrocytes in demyelination in this model.

### ADAM12 induces IFNγ^+^ Th1 cells to exacerbate neurological deficits in EAE

To determine the function of ADAM12 in the pathogenicity of CD4^+^ T cells in EAE, we measured CD4^+^ T cells among all CNS-infiltrating cells in EAE mice by flow cytometry. We observed a substantially higher percentage of CD4^+^ T cells in *ADAM12*^*+/+*^ compared with *ADAM12*^*−/−*^ mice (Fig. 4i).

Next, we studied the impact of ADAM12 on effector Th1 and Th17 cells. The ability of autoreactive T cells to induce autoimmunity and neuroinflammation is dictated by not only their specificity to self-antigens but also, more importantly, their effector functions. Thus, we examined the cytokines that were produced in the CNS tissues of EAE mice. By immunofluorescence staining, there were drastically fewer IFNγ^+^TCRα^+^ and IFN-γ^+^CD3^+^-infiltrating T cells in the spinal cords of *ADAM12*^*−/−*^ versus *ADAM12*^*+/+*^ mice (Fig. 4j, k). In contrast, no differences in IL-17^+^ TCRα^+^ T cells were found in the CNS tissue between groups (Fig. 4l, m), indicating the specific impact of ADAM12 on Th1 cells, rather than Th17 cells.

Antigen-specific encephalitogenic T cells are the main contributors to the induction of EAE.^8^ Thus, to determine whether the cytokine profile in the CNS reflects peripheral antigen-specific T-cell activation and effector function, we studied T-cell proliferation against the recall autoantigen MOG_35–55_ by BrdU assay. We noted a significant increase in MOG_35–55_-specific T-cell proliferation in *ADAM12*^*+/+*^ compared with *ADAM12*^*−/−*^ mice (Fig. 4n), despite no difference in splenocyte numbers (Supplementary Fig. 3a). Further, the production of soluble cytokines was evaluated on stimulation with MOG_35–55_. In addition to IFNγ and IL-17, the hallmark Th1 and Th17 cytokines, respectively (Fig. 4o, p), IL-6, IL-10, IL-13 (Th2), and TGF-β (FoxP3^+^ Tregs) were measured by ELISA (Supplementary Fig. 3b–e). IL-6 is produced by several cell types, including APCs, and promotes Th2 differentiation and simultaneously inhibits Th1 polarization.^25^ Moreover, IL-6 and TGF-β cooperate to induce Th17 cell differentiation.^26^ Nevertheless, only MOG_35–55_-specific IFNγ rose significantly in *ADAM12*^*+/+*^ versus *ADAM12*^*−/−*^ mice (Fig. 4o). These findings suggest that ADAM12 regulates CNS inflammation through the upregulation of autoreactive IFNγ^+^ Th1 cells.

Notably, a reciprocal relationship between ADAM12 and TGF-β has been reported, wherein ADAM12 stimulates TGF-β signaling, whereas TGF-β induces ADAM12 expression.^27^ Significantly, TGF-β alone induces the FoxP3^+^ T_reg_ transcription factor Foxp3 and is essential for the development of FoxP3^+^ T_regs_ in the periphery.^15^ Thus, we measured the expression of TGF-β and Foxp3^+^T_regs_ in *ADAM12*^*+/+*^ and *ADAM12*^*−/−*^ mice. We found no differences in antigen-specific TGF-β production in splenocyte culture supernatants by ELISA (Supplementary Fig. 3e). Moreover, the FoxP3^+^ T_reg_ population (Foxp3^+^CD4^+^ T cells) from the thymus (Thy), mesenteric lymph nodes (Mes), and spleen (SP) was analyzed by flow cytometry, and again, no differences were observed between *ADAM12*^*+/+*^ and *ADAM12*^*−/−*^ mice (Supplementary Fig. 3f). In addition to peripheral organs, there were no significant differences in FoxP3^+^T_reg_ cells among CNS-infiltrating cells from EAE mice between the *ADAM12*^*+/+*^ and *ADAM12*^*−/−*^ groups (Supplementary Fig. 3g). These results prompted us to conclude that ADAM12-induced EAE is independent of TGF-β production and FoxP3^+^ T_reg_ regulation.

We examined the possible function of ADAM12 in regulating a more recent subset of regulatory T cells with strong suppressive function in neuroinflammation: FoxA1^+^T_reg_ cells.^28,29^ However, we did not observe any difference in FoxA1^+^T_reg_ cells among CNS-infiltrating T cells (Supplementary Fig. 3h).

Collectively, our data show that ADAM12 is critical for antigen-specific Th1 responses during peripheral T-cell activation and thus their capacity to induce and sustain neuroinflammation in EAE. Conversely, the Th17-mediated arm of neuroinflammation is not influenced by ADAM12, and consequently, partial EAE developed in *ADAM12*^*−/−*^ mice. Moreover, the absence of *ADAM12* is not central in regulating CNS inflammation by impacting FoxA1^+^T_reg_ or FoxP3^+^T_reg_ numbers. Although we have specifically examined the function of ADAM12 in T-cell activation and effector functions with regard to EAE, our findings do not exclude the possibility that ADAM12 functions in other cells in immune organs or in CNS-resident cells.

### Antibody-induced ADAM12 costimulatory signaling in vivo leads to Th1 cell proliferation and promotes EAE

Genomic deletion of immune system genes can cause developmental defects in immune cells and initiate compensatory mechanisms. ADAM12 has been reported to be expressed by neurons, astrocytes, and oligodendrocytes in the CNS.^30^ Reactivation of autoreactive T cells in the CNS is important for the outcome and severity of EAE. To avoid this complexity and to examine the specific function of ADAM12 in initial autoreactive T-cell activation—i.e., in the early phase of the disease in the periphery—we administered ADAM12 Fab antibodies in vivo. Compared with the isotype control-treated group, the combination of 7B8 and 7G3 Fab induced markedly more severe EAE in the acute and chronic phases and during the entire disease duration, based on the area under the curve (Fig. 4q–s), supporting a role for ADAM12-mediated immune activation and disease induction with a subsequent impact on the outcome of the chronic phase.

Next, we examined whether anti-ADAM12 treatment in vivo affects the effector function and profile of autoreactive T cells in EAE. Splenocytes from EAE mice were cultured with MOG_35–55_, and Th1, Th2, and Th17 cytokine production was measured. Of the panel of 12 cytokines, IFNγ rose dramatically in the 7G3- and 7B8-treated group (Fig. 4t, u), indicating that anti-ADAM12 signaling triggered T-cell activation and IFNγ production in normally matured T cells. Further, IL-2 and TNFα, which are usually associated with Th1 cells, also increased (Fig. 4t). These data suggest that ADAM12 signaling in vivo triggers costimulatory signaling in antigen-specific Th1 cells, supporting our in vitro findings, thus functioning as a costimulatory molecule during Th1 cell activation and the pathogenicity of EAE.

### Transcriptomic profiling of *ADAM12*^−/−^ T cells reveals dysregulated costimulation, activation, and proliferation pathways

**Fig. 5:**
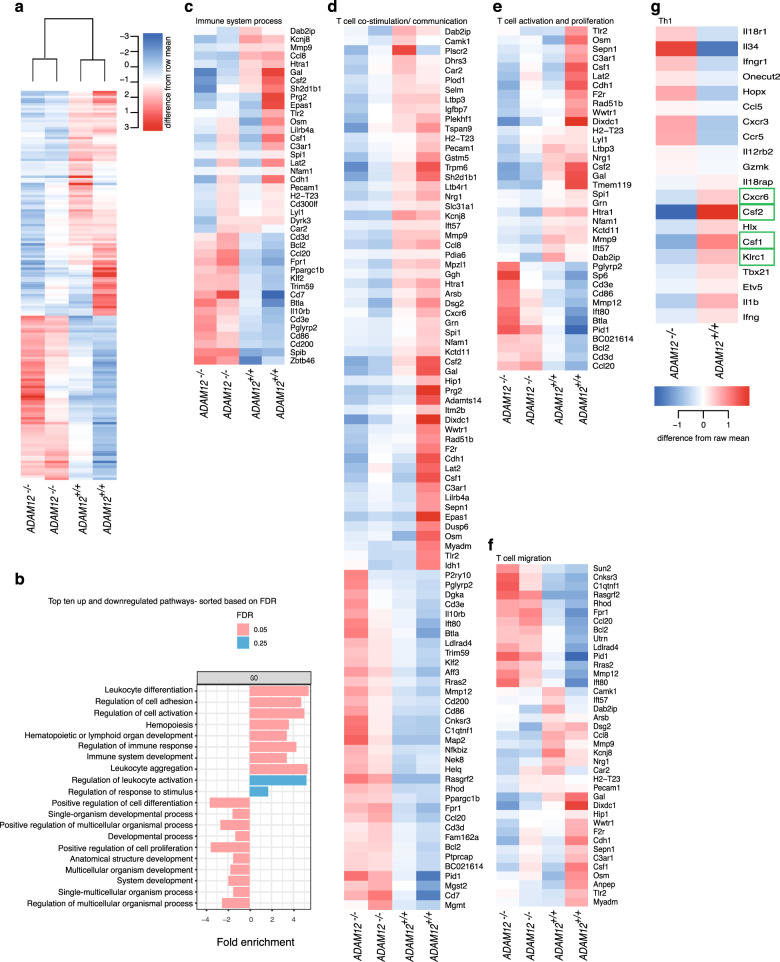
Transcriptomic profiling of *ADAM12*^*−/−*^ T cells reveals dysregulated T-cell costimulation, activation, and proliferation pathways. **a** A total of 161 differentially expressed genes (*P* < 0.05) in *ADAM12*^*−/−*^ versus *ADAM12*^+/+^ MOG_35–55_-reactive T cells, represented as a heatmap. The heatmap depicts two independent *ADAM12*^*−/−*^ samples, clustered apart from the two *ADAM12*^+/+^ replicates. Heatmaps of **b**: the top ten upregulated and downregulated pathways in *ADAM12*^*−/−*^ versus *ADAM12*^+/+^; the order is based on the FDR, and the *x* axis is the fold enrichment in the pathways. **c** Immune system process pathway (GO: 0002376, GO: 0002520). **d** T-cell costimulation/communication pathway (GO: 0048583, GO:0050896). **e** T-cell activation and proliferation pathway (GO: 0008283, GO: 0046649, GO: 0030098). **f** T-cell migration pathway (GO:0006928, GO:0032879). **g** Expression of Th1 signature genes in *ADAM12*^*−/−*^ versus ADAM12^+/+^ MOG_35–55_-reactive T cells

To verify the function of ADAM12 in T cells, we established the transcriptomic profile of in vivo-generated, antigen-activated T cells from *ADAM12*^*−/−*^ and *ADAM12*^*+/+*^ mice by RNA-sequencing (RNA-seq). We identified the gene signature of ADAM12 in T cells, comprising 161 significantly differentially expressed genes in *ADAM12*^*−/−*^ versus *ADAM12*^+/+^ T cells (Fig. 5a; Supplementary File 1) (*P*-value < 0.05, *n* = 161, downregulated: 93, upregulated: 68).

We then used the database for annotation, visualization, and integrated discovery (DAVID) tool to functionally annotate the differentially expressed genes. A total of 351 significant pathways (*P*-value < 0.05) were identified using GO Term biological processes (Supplementary File 2). Next, by functional annotation clustering, 97 clusters were formed, based on GO Term biological processes, among which 13 pathways with the highest number of overlapping gene profiles were selected that were included in the top 7 clusters (Supplementary Fig. 4a).

In addition, we used DAVID to generate upregulated and downregulated pathways. The top ten pathways are shown in Fig. 5b, and the list of all pathways with the corresponding FDR and *P*-values is provided in Supplementary File 3, which collectively established that T-cell activation, proliferation, costimulation, and migratory capacity are affected by the absence of *ADAM12*.

Immune system process was among the top differentially clustered pathways. This pathway harbored highly regulated genes that are associated with immune responses that are involved in activating cell surface receptor signaling pathways (Fig. 5c). Further, most of the remaining top pathways highlighted the importance of ADAM12 in T-cell costimulation/communication (Fig. 5d), T-cell activation and proliferation (Fig. 5e), and T-cell migration, wherein adhesion molecules and chemokine–chemokine receptors were overrepresented (Fig. 5f).

Notably, the transcriptomic profile of ADAM12-deficient T cells revealed that to compensate for the absence of the ADAM12 costimulatory signal and to promote T-cell activation and proliferation, these cells upregulated other costimulatory molecules, such as CD86/B7.2, that provide costimulatory signals that are necessary for T-cell activation and survival.^31^

Several of these compensatory genes that were upregulated in *ADAM12*^*−/−*^ T cells merit acknowledgment. CD200/OX-2 mediates a T-cell costimulatory signal that enhances T-cell proliferation.^32^ Btla is induced during T-cell activation, during which it acts as a inhibitory receptor for a B7-like ligand.^33^ *ADAM12*^*−/−*^ T cells also upregulated coreceptors of TCR activation, including CD3e and CD3d. These coreceptors are part of the TCR-CD3 complex, which couples antigen recognition to several intracellular signal transduction pathways during activation and effector function.^34^ *Cd7* was upregulated in *ADAM12*^*−/−*^ T cells and has been reported to mediate costimulation and T-cell signal transduction.^35^

Next, we analyzed Th1 and Th17 signature genes^36^ to establish any differences in *ADAM12*^*−/−*^ versus *ADAM12*^+/+^ T cells. Notably, by heatmap, Th1 surrogate marker genes, such as T-bet (*Tbx21*) and IFNγ (*Ifng*), were differentially regulated, specifically compared with other Th1 cytokines, such as GM-CSF (*Csf2*) and M-CSF1 (*Csf1*) (Fig. 5g). Further, the T-cell homing receptor *Cxcr6*, which also promotes IFNγ production,^37^ was significantly upregulated (green boxes in Fig. 5g; Supplementary Fig. 4b). However, although several Th17 genes were slightly differentially expressed (Supplementary Fig. 4c), no signature gene, including ROR γt (*Rorc*), was significantly different.

Notably, several upregulated genes in the T-cell activation/costimulation pathways, including *Nfkbiz*, have been reported to be important for helper T-cell generation and, simultaneously, Th17 function. Supporting these findings, mouse *Nfkbiz*^*−/−*^ T cells fail to generate Th17 cells, influencing the susceptibility to EAE.^38^ Moreover, *Btla*, which was upregulated in *ADAM12*^*−/−*^ T cells, has been shown to significantly suppress IFNγ production on overexpression. Vital factors in T-cell signaling pathways, including mitogen-associated protein kinases (MAPKs), nuclear factor-kappa B (NF-κB), and nuclear factor of activated T cells (NFAT), are also significantly repressed by overexpression of BTLA.^39^

The transcriptomic profile of *ADAM12*^*−/−*^ T cells strongly establishes that ADAM12 is a costimulatory molecule that cooperates with TCR signaling to trigger successful T-cell activation, proliferation, and Th1 cell fate and maintenance.

### *ADAM12*^−/−^ T cells are defective in inducing skin inflammation

**Fig. 6:**
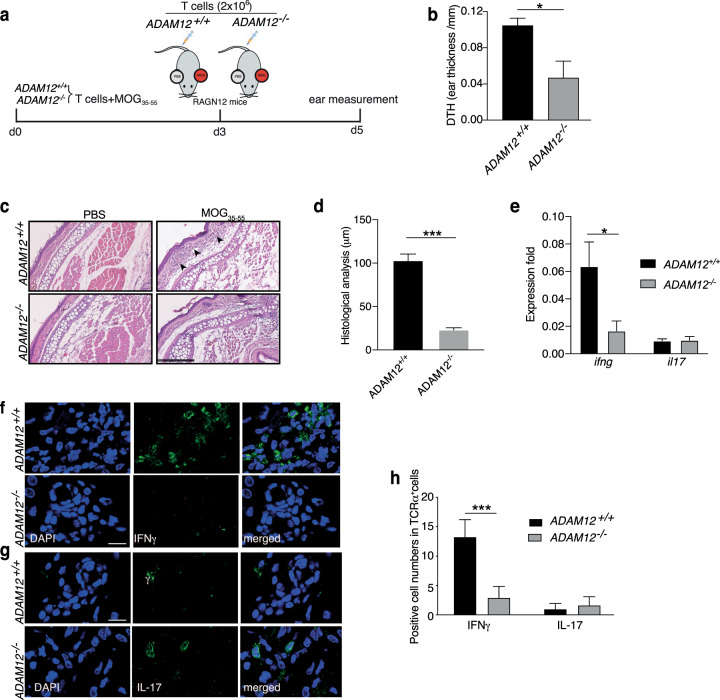
*ADAM12*^*−/−*^ T cells are defective in inducing skin inflammation. *ADAM12*^*+/+*^ and *ADAM12*^*–/–*^ spleens were taken from MOG_35–55_-induced EAE mice. Single-cell suspensions were established and treated with MOG_35–55_ (50 μg/ml) for 3 days. **a** Scheme shows an adoptive transfer model of DTH in Rag2^*−/−*^ immunocompromised mice. A total of 2 × 10^6^ MOG_35–55_-reactive *ADAM12*^*−/−*^ or *ADAM12*^+/+^ T cells were injected intravenously into mouse tail veins. Then, PBS or MOG_35–55_ was injected into the ears. **b** DTH reaction was measured by ear thickness after 48 h. Graphs are mean ± SEM; *N* = 4 mice per group. *P < 0.05 by Student’s *t* test. **c** H&E staining of ear tissue. Scale bars = 250 μm. **d** Histological analysis of ear skin thickness between MOG_35–55_-injected and PBS-injected animals. Graphs are mean ± SEM; *N* = 4 mice per group. ***P < 0.001 by Student’s *t* test. **e** Fold change in *ifng* and *il17* in ear tissue, compared with *gapdh* (equal to 1). Graphs are mean ± SEM; *N* = 3 mice per group from one experiment. *P < 0.05 by two-way ANOVA with post-Tukey’s multiple comparisons test. **f** Immunofluorescence staining of IFN-γ^+^ cells among ear-infiltrating cells: IFN-γ (green), and DAPI (blue); scale bars = 10 μm. **g** Immunofluorescence staining of IL-17^+^ cells among ear-infiltrating cells: IL-17 (green), and DAPI (blue); scale bars = 10 μm. **h** Quantification of ear-infiltrating cells from the immunofluorescence stains. ***P < 0.001 by two-way ANOVA with post-Tukey’s multiple comparisons test

Next, we determined whether the transcriptomic profile of antigen-driven *ADAM12*^*−/−*^ T cells is associated with their capacity to establish inflammation and is stable in vivo, using the delayed-type hypersensitivity (DTH) T-cell-mediated inflammatory model. To avoid any impact of ADAM12 expression by other immune or tissue cells, we established an adoptive transfer model of DTH in Rag2^*−/−*^ immunocompromised mice (Fig. 6a). A total of 2 × 10^6^ MOG_35–55_-reactive *ADAM12*^*−/−*^ or *ADAM12*^+/+^ T cells (established in parallel with those used for RNA-seq; Fig. 5) were transferred intravenously, and PBS or MOG_35–55_ was then injected into different ears. As a sham control, 2 × 10^6^ naive T cells were injected, which, as expected, did not elicit any inflammatory response, whereas MOG_35–55_-activated T cells caused inflammation of the ear (Supplementary Fig. 5a–d).

The adoptive DTH model allowed us to address the capacity of T cells exclusively to respond to the recall antigen on infiltrating the ear skin without the complexity that is often associated with EAE models. EAE is a highly complex neuroinflammatory model that requires both T-cell activation in the periphery and the ability to cross the specialized endothelial cell layer of the BBB. Encephalitic T cells must overcome the glial limitans and be reactivated by professional APCs, including activated CNS local glial cells in the parenchyma, to determine the neuroinflammatory outcome.

We found that *ADAM12*^*−/−*^ T cells, in contrast to *ADAM12*^+/+^ T cells, were defective in provoking skin inflammation, as evidenced by their significantly lower ear thickness (Fig. 6b), and decreased T-cell infiltration, based on histology of the skin by H&E staining and quantification of histology score (Fig. 6c, d). Moreover, there was a dramatic difference in *Ifng* mRNA and infiltrating IFNγ^+^ T cells into the ear tissue between *ADAM12*^*−/−*^ and *ADAM12*^+/+^ animals, but no differences were found for *Il17* mRNA, associated with equal infiltration of few IL-17^+^ T cells in both groups (Fig. 6e–h). Consistent with their transcriptomic profile, *ADAM12*^+/+^ T cells were able to establish Th1-driven skin inflammation.

These results conclusively establish an important function for ADAM12 in T cells in costimulation, the response to antigenic activation, IFN-γ production, and the ability to cause and sustain tissue inflammation.

## Discussion

The immune system has the specialized ability to defend against a wide range of microbial pathogens and remove the subsequent damaged self-tissue, without responding to self-antigens. The discovery of the TCR provided an explanation for the specificity and diversity of T-cell responses. Yet, functional studies revealed that antigen alone was not sufficient to drive the activation of naive T cells, leading to the two-signal concept of T-cell activation. Accordingly, productive T-cell activation requires an initial signal that is supplied by the interaction of antigenic peptide/major histocompatibility complex with the TCR and a second, antigen‐independent costimulatory signal.^5^ Since the discovery of several costimulatory signaling molecules, including inhibitory factors, some have become active targets for immunotherapies in autoimmune diseases,^40^ transplantation,^41^ and cancers.^42^

ADAM12 has been reported to be involved in many biological functions, primarily the regulation of cell adhesion, differentiation, proliferation, and survival of various cancers.^43^ However, there are few functional studies on ADAM12 in T cells and in the regulation of immune responses. In this regard, an initial report showed that ADAM12 mRNA was upregulated in T cells that infiltrate the CNS in EAE mice,^11^ suggesting that ADAM12 mediates the migratory capacity of T cells. A more recent study found that ADAM12 mRNA is highly expressed in FoxP3^+^ T_regs_ and Th17 cells, implicating a connection with TGF-β signaling in human peripheral blood mononuclear cells (PBMCs).^14^ However, no previous study has addressed the function of ADAM12 directly in T cells in general or in any model of tissue inflammation.

Here, we determined the function of ADAM12 in T-cell activation, immune responses, and its impact on T-cell-mediated functions in conducting tissue inflammation. Based on the limited literature, our main hypothesis was that ADAM12 is an adhesion molecule that promotes the T-cell capacity to migrate and infiltrate tissues. Notably and unexpectedly, we found that ADAM12 functions as a costimulatory signaling molecule on T cells that is important for amplifying TCR signaling and stimulates T-bet-mediated IFNγ production, thus directing the outcome of two tissue-specific inflammatory models: neuroinflammation in EAE and skin inflammation in DTH.

We found that although at the mRNA level *Adam12* is much lower in T cells than *Cd28*, ADAM12 protein is expressed by up to 44% of CD4^+^ T cells. However, in vitro and in vivo activation of T cells led to significant downregulation of ADAM12 protein. Our subsequent examination revealed that ADAM12 on T cells triggers a costimulatory signal that is similar in intensity to CD28 in vitro, in both human and murine T cells. ADAM12 synergizes with combined CD28/CD3-induced activation of T cells or replaces CD28 and triggers CD3/ADAM12-mediated T-cell activation. Accordingly, treatment with activating ADAM12 antibodies with anti-CD3 or in combination with anti-CD3/anti-CD28 led to significant amplification of TCR signaling (based on pZAP-70 levels), upregulation of the activation marker CD69, and proliferation of purified CD4^+^ T cells.

Human ADAM12 is expressed as two isoforms, the prototypical transmembrane ADAM12-L and a short, secreted version, ADAM12-S, which lacks the cytoplasmic tail and transmembrane domain. In mice, only ADAM12-L is expressed.^21^ This difference indicates additional complexity by which ADAM12 activates T cells in human versus mouse, given that ADAM members have both adhesive and proteolytic activities.^43^ To determine whether the costimulatory function of ADAM12 is mediated by the enzymatic activity of ADAM12-S, we administered enzymatically active recombinant ADAM12-S with anti-CD3 to purified human CD4^+^ T cells. However, ADAM12-S was unable to trigger a T-cell costimulatory signal that led to T-cell proliferation.

Because different substrates are cleaved by the two forms of ADAM12 in human,^21^ the impact of the proteolytic activity of transmembrane ADAM12 (ADAM12-L) on cell proliferation was further evaluated. Although mutant ADAM12-L lacked proteolytic function, wild-type and mutant ADAM12-L induced similar levels of cell proliferation. These results demonstrate that the ADAM12-induced T-cell costimulatory signal is not mediated by its cleavage of other molecules but exerts its function directly by triggering a costimulatory signal.

Moreover, to verify that the impact of the antibodies was not due to off-target effects, we examined T cells from *ADAM12*^*−/−*^ and *ADAM12*^+/+^ mice. The combination of anti-ADAM12 and anti-CD3 amplified TCR signaling (based on pZAP-70 levels) and thus the proliferation of *ADAM12*^+/+^ T cells, whereas *ADAM12*^*−/−*^ T cells were deficient responders to anti-ADAM12/anti-CD3 signaling. Further analysis revealed that ADAM12 costimulatory signaling influences IFNγ production exclusively, having no influence on other T-cell cytokines, particularly IL-17. This latter finding was unexpected, in light of the lone study that showed that ADAM12 mRNA is highly expressed in Th17 cells.^14^ We speculate that the genomic deletion of ADAM12 affects general immune responses, which could explain the significant lack of IFNγ production by *ADAM12*^*−/−*^ T cells.

To understand how ADAM12 governs various CD4^+^ T cells subsets without influencing their thymic development—or has indirect affects via APCs—we knocked down ADAM12 in purified naïve CD4^+^ T cells by siRNA. ADAM12 knockdown prior to differentiation of Th1 cells abolished T-bet expression and IFNγ production. Notably, although it did not alter the pattern of RORγt expression or IL-17 production, ADAM12 knockdown prior to Th17 cell polarization again suppressed T-bet and thus reduced T-bet^+^RORγt^+^ double-positive cell numbers and IFNγ production. Moreover, *ADAM12* deficiency and ADAM12 knockdown did not affect FoxP3^+^T_regs_. Future studies should determine the ligand for ADAM12 to better understand the molecular mechanisms that lead to its T-cell costimulatory signal and the Th1 cell fate.

In CNS diseases, ADAM12 has been reported to be responsible for impairing the neural vascular barrier that is induced by hypoxia in the brain.^44^ However, the function of ADAM12 in autoimmune-mediated CNS inflammatory diseases has not been studied extensively. Here, we found that mice that lack ADAM12 develop significantly lower MOG_35–55_-specific T-cell responses and thus significantly less severe clinical symptoms of EAE. Although our results do not exclude the possibility that ADAM12 functions in the neural vascular barrier or in CNS-resident cells, our further study revealed that ADAM12 is distinctly associated with T-cell activation and Th1 responses, wherein the significantly lower IFNγ production in MOG_35–55_-specific T cells in the periphery correlated directly with reduced parenchymal infiltration of IFNγ^+^CD3^+^ T cells and neuroinflammation.

Because ADAM members also have proteolytic activities and regulate cell adhesion and thus the migration and metastatic activity of various cancers,^43^ we speculated that ADAM12, in addition to activating Th1 cells in the periphery, accelerates T-cell infiltration into the CNS to establish neuroinflammation in EAE. Despite careful examination, although we noted fewer cells in vessels, their number in the parenchyma did not differ significantly. Also, whereas we observed a significantly lower number of IFNγ^+^CD3^+^ T cells, we did not find differences in IL-17^+^CD3^+^ T cells in the CNS. This stark evidence suggests that the absence of ADAM12 impairs Th1 cell activation and likely their migratory capacity through the BBB. This concept was further supported by our finding that Cxcr6, a proposed Th1 homing chemokine receptor,^37^ was significantly downregulated in *ADAM12*^*−/−*^ T cells. However, *ADAM12*^*−/−*^ T cells did not show general defects in migration.

To exclude the possibility of compensatory mechanisms or developmental effects of a genomic lack of *Adam12*, thus dismissing a function for ADAM12 in other CNS-resident cells, and to determine the function of ADAM12 in vivo during the peripheral activation of encephalitogenic T cells, we treated mice with anti-ADAM12 Fab fragments on days 0 and 7 of EAE induction. We used Fab fragments to prevent activation of the complement system and FcgR interactions, which can interfere with general peripheral immune activation. We observed that EAE was exacerbated in animals that received anti-ADAM12 compared with the control antibody- and PBS-treated groups. Accordingly, positive induction of ADAM12 signaling was associated with elevated peripheral MOG_35–55_-specific T-cell responses, reflected by significant IFNγ production. These results support our in vivo findings in *ADAM12*^*−/−*^ EAE mice.

Whereas we observed highly supportive evidence for the function of ADAM12 in activating Th1-mediated immune responses, both in vitro and in vivo, it was unexpected that we did not find any correlation with Th17-mediated responses or FoxP3^+^T_regs_. Accordingly, we did not detect any differences between groups regarding TGF-β production. TGF-β is a powerful inducer of FoxP3^+^T_regs_^15^ and thus relevant in suppressing EAE.^19^ Moreover, TGF-β has an important function in the differentiation of Th17 cells.^16^ Previous research has shown that ADAM12 interacts with TGF-βRII to enhance TGF-β signaling,^27^ prompting an earlier study of TGF-β signaling with regard to ADAM12 in human PBMCs. The group showed that in purified FoxP3^+^T_regs_ and CCR6^+^ Th17 cells, ADAM12 mRNA was highly expressed. However, knockdown of *Adam12* led to an increase in IL-17,^14^ indicating that ADAM12 is not necessary for Th17 cells but suppresses IL-17 production instead.

We then examined whether a lack of ADAM12 increased the number of a more recently described suppressive regulatory T-cell type, FoxA1^+^T_regs_,^28,29^ to explain the less severe CNS inflammation during EAE. Yet, we did not observe any alterations in CNS-isolated FoxA1^+^T_regs_ in *ADAM12*^*−/−*^ mice, indicating that ADAM12 is dispensable for FoxP3^+^T_reg_ and FoxA1^+^T_reg_ generation.

To determine the general function of ADAM12 in T cells, we generated transcriptomic profiles of *ADAM12*^+/+^ versus *ADAM12*^*−/−*^ T cells, which revealed significantly differentially expressed genes that clustered in pathways with overlapping functions. These unbiased findings solidified our observations that ADAM12 is indeed vital in regulating T-cell responses to stimuli, T-cell communication and costimulatory signaling, T-cell activation and proliferation, and migration. Moreover, a qualitatively different Th1 signature was observed between *ADAM12*^+/+^ and *ADAM12*^*−/−*^ T cells, including lower T-bet (*Tbx21*) and IFNγ (*Ifng*) levels, whereas there was no significant difference in any Th17 signature gene. Although the relative contribution of IFNγ to neuroinflammation is debated,^45^ it has recently been suggested that T-cell expression of T-bet, the hallmark Th1 transcription factor, defines the pathogenicity of not only Th1 cells but also Th17 cells.^46^ Accordingly, the inhibition of T-bet has been shown to ameliorate EAE by inhibiting Th1 and Th17 cells.^24^

Further, four genes in the Th1 signature were significantly differentially expressed: *Klrc1*, *Cxcr6* (the homing chemokine receptor of Th1 cells^37^), *Csf1* (M-CSF), and *Csf2* (GM-CSF). Notably, GM-CSF-deficient mice are resistant to EAE.^47^ Emerging evidence suggests that GM-CSF is involved in autoimmune diseases.^48^ In MS, GM-CSF has been suggested to define a T-helper cell signature.^49^ In support of our findings that the lack of *Adam12* results in fewer Th1 cells that express GM-CSF while failing to influence Th17 cells, it has been reported that in human T cells, IL-17 and GM-CSF expression is antagonistically regulated by human T-helper cells, wherein GM-CSF is associated with the Th1 axis.^50^

In support of our transcriptomic data, on adoptive transfer, in vivo-activated *ADAM12*^+/+^ T cells induced skin inflammation in a DTH model and maintained their IFNγ-producing capacity, reinforcing their Th1-mediated pathogenicity. In contrast, *ADAM12*^*−/−*^ T cells failed to induce or sustain Th1-mediated skin inflammation.

Our data collectively establish that ADAM12 is a T-cell costimulatory molecule that is required to amplify TCR signaling for robust T-cell activation and T-bet-mediated IFNγ production (for Th1 cell generation and function), which in turn are pivotal for the outcomes of skin and CNS inflammation (Supplementary Fig. 6). Given that disparate immune responses (Th1 vs. Th17) are associated with different categories of autoimmune disease—and even between subsets of patients in a specific autoimmune disease, such as in MS^51^—future studies should determine whether ADAM12 is a potential target that represses tissue-specific inflammation, particularly in those with a Th1-mediated disease. These novel results highlight unmet needs in provoking Th1-mediated immune responses to combat various cancers.

## Materials and methods

### Mice

ADAM12^*−/−*^ mice, also known as meltrin alpha knockout mice,^52^ were backcrossed with C57BL/6 mice. Littermates were bred and kept in conventional animal facilities at the University of Copenhagen; 8–12-week-old *ADAM12*^+/+^, and *ADAM12*^*−/−*^ littermates were used to induce EAE and C57BL/6 for EAE with antibody treatment. Rag2^*−/−*^/RAGN12 (NM_009020) and SCID mice were purchased from Taconic (Taconic, Bioscience).

All experiments were performed in accordance with the ethical committees in Denmark (2013–15–2934–00807, 2018–15–0202–00123, and 2018–15–0201–01573) and approved by institutional review boards.

### Induction of experimental autoimmune encephalomyelitis (EAE)

Active EAE was induced with MOG_35–55_ as described.^53^ C57BL/6 *ADAM12*^+/+^ and *ADAM12*^*−/−*^ littermates were immunized with 150 µg MOG (35–55) peptide in CFA-H37Ra (Difco) subcutaneously (s.c.) at the base of the tail, followed by a single dose of pertussis toxin (Sigma) (400 ng in PBS) intraperitoneally (i.p.) on day 0. The mice were screened for EAE symptoms and sacrificed on day 30. EAE was scored clinically as follows: 0—no disease; 1—limp tail; 2—limp tail and ataxia and hind limb weakness/unsteady walk; 3—1 hind limb affected; 4—2 hind limbs affected; 5—complete paralysis of hind limbs until hips; and 6—moribund or dead. If the mice reached a score of 5 and failed to improve within 48 h or lost over 25% of their weight, they were euthanized.

In certain experiments, mice were injected with anti-ADAM12 F(ab) fragments of monoclonal antibodies (7B8 and 7G3) against human ADAM12, which were generated by immunizing mice with full-length recombinant human ADAM12. The immunogen was produced in 293 cells, purified, and used to immunize mice. Polyclonal antisera to human ADAM12 included rb122, raised against the recombinant cysteine-rich domain, and rb134, raised against purified full-length recombinant ADAM12, as described.^18,54^

Fab fragments were prepared using the Pierce™ Fab preparation kit (Cat:44985, ThermoFisher) and injected into mice at 200 μg/mouse. This dose corresponds to 10 mg/kg (the average weight of a mouse is 20 g), based on reported antibody treatments in vivo.^55,56^ By ELISA-based detection of antibodies in the serum of mice that were injected with them, the half-life of the ADAM12 antibodies was ~3 days; thus, each mouse received two injections, on days 0 and 7 post immunization, to encompass the T-cell activation phase.

### Preparation of CNS-infiltrating cells

At the indicated times after active EAE was inducted, the brain and spinal cord were removed immediately. The tissues were homogenized, and lymphocytes were obtained using a Percoll gradient.^19^

### EAE CNS tissue preparation and Luxol fast blue/hematoxylin/eosin staining (LHE staining)

All EAE experiments were terminated on approximately day 30 post immunization. Four to six CNS tissues per group were prepared for histological and pathological analysis. The mice per group were selected to represent the clinical severity of EAE in the group.

Cryosectioned CNS and paraffin-embedded ear tissues were processed for Luxol fast blue staining and measured as described.^53^

### Analysis of LHE-stained CNS tissues by light microscopy

Vessels and small and large infiltrates per tissue slice were counted at 100× magnification. To further analyze tissue infiltration near vessels, images at 2576 × 1932 pixels were taken under 200× magnification. Using Corel Photo-Paint X3, two circles were drawn around each vessel, with a diameter of 209 and 697 pixels, respectively. The number of infiltrates inside of the small circle and inside of the large but outside of the small circle was determined (see Supplementary Fig. 2A). In certain cases, the larger circle contained infiltrates that obviously stemmed from a neighboring vessel, which were not counted. In addition, groups of closely neighboring vessels and vessels near the edges of the tissue or central canal were disregarded.

### Establishment of MOG-specific encephalitogenic Th1 cell lines

To generate T-cell lines, 8–12-week-old C57BL/6 mice were immunized in the flank and tail base with 200 μl of a 1:1 emulsion of 150 μg MOG_35–55_ in PBS and CFA, containing *Mycobacterium tuberculosis* H37Ra (Difco). Draining lymph nodes were collected 10 days after immunization, and a single-cell suspension was prepared as described.^19^

### Adoptive transfer of delayed-type hypersensitivity

*ADAM12*^+/+^ and *ADAM12*^*−/−*^ mice were immunized with MOG_35–55_ as described for EAE, spleens were harvested, and single-cell suspensions were established (3 × 10^6^/ml) and treated with 50 μg/ml MOG_35–55_ for 3 days. Antigen-specific *ADAM12*^+/+^ and *ADAM12*^*−/−*^ T cells were then harvested, and 2 × 10^6^ of each T-cell type in 150 μl of PBS were injected into 8-week-old Rag^*−/−*^/RAGN12 mouse via the tail vein. One hour later, the RAGN12 mice received 500 μg of MOG_35–55_ in 100 μl of PBS as recall antigen via subcutaneous injection of the ear. PBS was injected as a negative control in the contralateral ear. The sham group received 2 × 10^6^ naive T cells, and were then challenged as above. After 48 h, the DTH response was measured as the difference in thickness (mm) of the ears between MOG_35–55_-injected and PBS-injected animals.

Ear tissue was stained with H&E and analyzed in VDP.view2 to measure skin thickness.

### Preparation of healthy human donor blood PBMCs

Blood donor buffy coats were used to prepare lymphocytes as described.^29^

### CFSE labeling

To assay T-cell proliferation, cells were labeled with CFSE (Molecular Probes) as described.^29^

### Sorting of T cells by MACS

Naive CD4^+^ T-cell Isolation Kits for mouse (130–104–453) and human (Cat. 130–094–131, Miltenyi Biotec) were purchased from Miltenyi Biotec and used per the manufacturer’s instructions.

### Proliferation analysis by BrdU incorporation

Animals were sacrificed on Day 30 ± 5 post immunization, and splenocytes were treated with MOG_35–55_ at 50 μg/ml to measure antigen-induced T-cell proliferation and cytokine production. Cell proliferation was measured using the BrdU Cell Proliferation Assay Kit (K306–200, Biovision).

In certain experiments, mouse T cells were activated with anti-CD3 (5 μg/ml; clone 145-C11, BD) and soluble anti-CD28 (2 μg/ml; clone 37.51, BD); human CD4^+^ T cells and Jurkat cells were treated with ImmunoCult™ Human CD3/CD28 T Cell Activator (Cat. 10991, STEM CELL technologies) prior to the BrdU cell proliferation assay.

### ELISA

Mouse IL-13 (88–7137–22), mouse IL-6 (88–7064–22), mouse IFN gamma (88–7314–22) ELISA kits were purchased from eBiosience. The mouse/rat/porcine/canine TGF-beta1 Quantikine (MB100B), mouse IL-17 Quantikine (M1700), mouse IL-10 Quantikine (M1000B), and human IFN gamma (DY285B) ELISA kits were purchased from R&D Systems. Mouse Th1/Th2/Th17 Cytokines Multi-Analyte ELISArray™ Kits (MEM-003A) were obtained from Qiagen. ELISAs were performed per the manufacturers’ instructions.

### FACS staining

FACS staining was conducted as described^29^ using the following antibodies: anti-mouse CD4 (L3T4, BD), anti-mouse TCR (553169, BD), anti-FoxP3 (17–5773–82, eBioscience), anti-T-bet (25–5825–82, eBioscience), anti-RORγt (17–6981–82, eBioscience), anti-CD69 (15–0691–82, eBioscience), pZAP-70 (557817, BD), anti-Ki-67 (612472, BD), and anti-ADAM12 7B8 (in-house).

Cells were acquired using FACSDiva after the exclusion of duplets. Dead cells were discriminated in all stains using the LIVE/DEAD Fixable Dead Cell Stain Kit at 405-nm excitation (L34955, Invitrogen). FlowJo 10.5.3 (Flowjo, LLC) was used for further analysis.

### siRNA silencing

Accell SMART pool small-interfering RNA was purchased from Dharmacon (cat: E-043494–00, Thermo Scientific), and was introduced into primary T cells according to the manufacturer’s protocol.

### Th1/Th17 polarization in vitro

Naïve CD4 T cells were isolated from spleens using the Naive CD4^+^ T Cell Isolation Kit (130–104–453, Miltenyi Biotec). Next, 96-well plates were activated overnight with plate-bound mouse anti-CD3 at 5 μg/ml (BD Pharmingen Clone 145-C11 cat. no. 553058) and mouse anti-CD28 at 2 μg/ml (BD Pharmingen Clone 37.51 cat. no. 553295) at 4 °C. Purified CD4^+^ T cells (2 × 10^5^) were cultured 200 μl cDMEM in the activated plate. To induce Th1 polarization in vitro, the media was supplemented with anti-IL-4 (10 μg/ml) (ebioscience clone 11B11 cat. no. 16–7041–85) and IL-12 (20 ng/ml) (R&D Systems cat. no. 419-ML-010). Th1 cells were used at 4–6 days polarization.

For Th17 polarization, T-cell media was supplemented with Th17-polarizing cytokines: IL-23 (10 ng/ml, Protein R&D Systems, 1886-ML), IL-6 (100 ng/ml, Protein R&D Systems, 406-ML/CF), rTGF-β1 (2 ng/ml, Protein R&D Systems, 7666-MB-005), anti-IL-4 (10 μg/ml, eBioscience, 16–7041–85), and anti-IFN-γ (10 μg/ml, eBioscience 16–7312–85). Th17 cells were ready for analysis on day 5 of polarization.

### Quantitative PCR

RNA was extraction using the RNeasy Mini Kit (#74104, QIAGEN), and quantitative PCR was performed with primers for *cd28 (*NM_007642, QIAGEN)*, ifng* (NM_008337, QIAGEN), *cd274* (NM_021893, QIAGEN), *il17(*NM_010552, QIAGEN), *adam12* -forward (CAC ACG GAT CAT TGT TAC TAC CA), *adam12* -reverse (ATT GGC TCT AAG CTG TAC GTT TT) (TAG Copenhagen A/S), and gapdh (NM_008084. QIAGEN) as a housekeeping control.

### Shedding assay

Plasmid: The wild-type and catalytically inactive (E351-A point mutation) full-length human ADAM12-L constructs in pcDNA3.1 have been described.^54^ The cDNA construct that encoded AP (alkaline phosphatase)-conjugated pro-EGF in the pRC/CMV vector (AP–EGF) was provided by Dr. Shigeki Higashiyama (Ehime University Graduate School of Medicine, Ehime, Japan).

ADAM12-mediated AP–EGF shedding was determined by co-transfection of Jurkat cells with wild-type or catalytically inactive (E351-A) full-length ADAM12-L, with AP–EGF, as described.^57^ At 48 h after transfection, cells were seeded into 24-well plates and, on the following day, were washed twice with SFM (serum-free medium). For photometric quantification of AP–EGF shedding, cell-conditioned medium was harvested, and the cell layer was lysed in 250 μl 1% Triton X-100 in PBS. Conditioned SFM or cell lysate (50 μl) was mixed with 50 μl of a 2-mg/ml solution of the AP substrate 4-nitrophenyl, and each well was assayed in duplicate in a 96-well plate. After incubation at 37 °C for 1–2 h, the amount of product was quantified by measuring the absorbance at 405 nm. All treatments were performed in triplicate. AP–EGF shedding in cells that were transfected with wild-type or catalytically inactive ADAM12 was calculated as the activity of AP in the conditioned medium divided by that in the medium and corresponding cell lysate, after subtracting the background signal from nontransfected cells.

### Immunofluorescence (IF)

Spinal cords of mice with EAE were dissected. Tissues were embedded in OTC compound (Sakura Finetek Denmark ApS, Værløse, Denmark) and snap-frozen in isopentane on dry ice. Tissues were sectioned into 6–10-μm slices, fixed in 4% paraformaldehyde (PFA) for 10 min, and stained with various antibodies. The primary antibodies for IF were: rabbit anti-CD3 (Abcam, ab16669), anti-TCRα (Abcam, Ab18861), goat anti-rabbit IgG-AF488 (Invitrogen, A11008), rat anti-IFNγ (BD, 554409), rat anti-IL-17 (BD, 555067), and goat anti rat-AF568 (Invitrogen, A11006). DAPI-Pacific blue (Invitrogen, D3571, 1:30000) was used to visualize nuclei. IF images were viewed under a Zeiss fluorescence microscope and a Leica Sp-8 confocal microscope.

### Image quantification

To count infiltrating cells, the areas of infiltration were selected manually, and a customized macro for counting cells was run, generating an automatic cell count.^28^

### Preparation of RNA-seq libraries

As described above, *ADAM12*^+/+^ and *ADAM12*^*−/−*^ mice were immunized with MOG_35–55_, and MOG_35–55_-reactive T cells were established by reactivating the T cells ex vivo for 3 days. The total RNA was extracted from MOG_35–55_-reactive *ADAM12*^+/+^ and *ADAM12*^*−/−*^ T cells (two mice per group) using the RNeasy mini kit (Qiagen, cat. no. 74104). mRNA was prepared using oligo-dT primers and reverse-transcribed into cDNA (KAPA Biosystem, cat. no. KK2601). The resulting dsDNA was tagmented (Illumina, cat. no. FC-131–1096) and then ligated with Illumina sequencing adaptors (Illumina, cat. no. FC-131–2002). The detailed protocol is described elsewhere.^58^

Libraries were pooled (1.6 pM) and sequenced as paired-end 75-bp reads on an Illumina NextSeq500. Biological duplicates were run for each sample. Preprocessing steps were performed using Fastq Screen, FastQC, and Trimmomatic. The sequencing reads were mapped to the Mus musculus genome (version mm10) with STAR (version 2.6.0b-2). There were between 12.3 and 14.5 M reads that were aligned per library. Read counts for annotated features were computed by htseq-count using the mm10 Mus musculus gene annotation from iGenome. Pairwise comparison between two conditions using their biological replicates was performed using DESeq2. All analyses were conducted using local Galaxy (BricwebGalaxy v19.05).

### Functional annotation

Differentially expressed genes between *ADAM12*^*−/−*^ versus *ADAM12*^+/+^ cells were selected (*P*-value < 0.05, *n* = 161, downregulated: 93, upregulated: 68) and the DAVID tool was used to assign functional annotations to them.^59^ From the top functional annotation clusters, several pathways with higher numbers of genes were selected.

Because most T-cell-associated genes were shared between related pathways, we combined the genes in related pathways and named them accordingly. These pathways included: Immune system process (GO: 0002376, GO: 0002520), T-cell costimulation/communication (GO: 0048583, GO:0050896), T-cell activation and proliferation (GO: 0008283, GO: 0046649, GO: 0030098), and T-cell migration (GO:0006928, GO:0032879). Heatmaps were prepared using Varistran in R.

### Statistical evaluations

Statistical analyses were performed using GraphPad Prism (GraphPad Software Inc.). Nonparametric Mann–Whitney and Kruskal–Wallis tests were used for comparisons between two and more than two groups, respectively. Student’s *t* test and one-way ANOVA were used to compare the FACS data between two groups and more than two groups, respectively. Two-way ANOVA with post-Tukey’s multiple comparison test was used to evaluate two factors in two-group comparisons. A value of *P* < 0.05 was considered to be significant.

Detailed statistical methods are indicated in the figure legends.

## Supplementary information

Supplementary Figure Legends

Supplementary Figure 1

Supplementary Figure 2

Supplementary Figure 3

Supplementary Figure 4

Supplementary Figure 5

Supplementary Figure 6

Supplementary File 1

Supplementary File 2

Supplementary File 3

## Data Availability

The RNA-seq data have been deposited under SRA accession number PRJNA636129. The datasets that were analyzed in this study are available from the corresponding author on reasonable request.
