# Supplementary material for: ADAM12 is a costimulatory molecule that determines Th1 cell fate and mediates tissue inflammation
Source: Cell Mol Immunol. 2020 Jun 22;18(8):1904–19. doi: 10.1038/s41423-020-0486-8 (PMC8322154; doi:10.1038/s41423-020-0486-8)
Supplement: Supplementary file 1 — Supplementary Figure Legends [file 41423_2020_486_MOESM1_ESM.docx]

Supplementary Figure legends

**Fig. S1** **ADAM12 is essential for T-bet expression in T cells and thereby IFNγ production**

**a**) Normalized *cd28 and adam12* expression in mouse spleen cells by qPCR. Data are from three independent samples (N = 3), Student *T* test was used, **p* < 0.05. **b**) Normalized *cd28, cd274 (pdl1) and adam12* expression in mouse CD4^+^T cells by qPCR. Data are from three independent samples (N = 3), One-way ANOVA with post Tukey's multiple comparisons test were used respectively, ****p* < 0.001. **c**) Normalized *adam12* expression in mouse spleen and CD4^+^T cells by qPCR. Data are mean±SD from three independent samples (N = 3), Student *T* test was used, **p* < 0.05. **d**) Normalized *cd274 (pdl1)* and *adam12* expression in mouse CD4^+^T cells by qPCR. Data are mean±SD from three independent samples, Student *T* test was used, **p* < 0.05. **e**) Jurkat cells were co-transfected with Wild-type and catalytically inactive (E351Q point mutation) full-length human ADAM12-L constructs in the pcDNA3.1 vector and AP–EGF vector for 48 hours, cells were seeded into 24-well plates and the following day washed twice with SFM (serum-free medium). For photometric quantification of AP–EGF shedding, both cell medium and cell lysed were harvest. AP–EGF shedding in cells was calculated as AP activity in the conditioned medium divided by AP activity in the medium and corresponding cell lysate after subtracting the background signal from non-transfected cells. Human CD4^+^ T-cells were purified from healthy donors and seeded on plate-bound anti-CD3 (5μg/ml) and soluble anti-CD28 (2μg/ml) for 6 days, anti-ADAM12 antibody 7G3 (10μg/ml, full antibody or Fab), 7B8 (10μg/ml, full antibody or Fab) were added to the cultures separately. Cell culture supernatants were collected for ELISA assays. Data are mean ± SEM from replicates in one experiment, N = 3, One-way ANOVA with post Tukey's multiple comparisons test were used respectively, **p* < 0.05, ***p* < 0.01. **f**) IFN-γ; Data are mean ± SEM, N = 9, One-way ANOVA with Bonferroni's multiple comparisons test was used, **p < 0.01, ***p < 0.001. **g**) IL-10; **h**) TGF-β1. **i**) ADAM12^+/+^ and ADAM12^–/–^ T cells were treated with plate-bound anti-CD3 (5μg/ml) and anti-ADAM12 antibody (10μg/ml) for 3 days. Cell culture supernatants were collected for a multiple-cytokine’s ELISA assay from three independent experiments. **j**) MOG_35-55_ –specific T cells were treated with MOG_35-55_ (50μg/ml), IL-12 (20ng/ml), anti-IL-4 (10μg/ml) and with siRNAs (siControl or siADAM12) application for 72 hours. Representative FACS plots show knockdown of ADAM12 efficiency. **k**) Representative FACS plots show CTLA-4, B7.1, PD-1, FoxP3, RORγt and FoxA1 expression. Graphs are representative from two independent experiments. **l**) Representative FACS plots show the gating strategy and T-bet, RORγt stained CD4^+^ T-cells. **m**) Quantification of FACS results from one representative data from two independent experiments. Graphs are mean ± SEM of triplicates, N = 3. Two-way ANOVA with post Tukey's multiple comparisons test were used respectively, ****p* < 0.001.

**Fig. S2** **ADAM12 is regulating the severity of neurological symptoms of EAE**

30 days after immunization for EAE. **a**) Analysis of frozen mouse spinal cord lumbar tissue. Representative pictures of the big (green arrows) and small (red arrows) circles that were used to

analyze the extravasation activity, scale bar= 250μM. **b**). Percentage of demyelination from luxol fast blue staining of spinal cord tissue. The total demyelinated area was then divided by the total area of the slice, to obtain the percentage of demyelination. Data are mean±SEM, N = 5 mice /group.

**Fig. S3** **ADAM12 induces IFNγ^+^Th1 cells to exacerbate EAE-neurological deficits**

CNS tissue and spleens were taken from EAE mice. **a**) Cell counting from splenocytes. Splenocytes were cultured with MOG_35-55_ (50μg/ml) for 48 hours, cell culture supernatants were collected for ELISA. **b**) IL-6; **c**) IL-10; **d**) IL-13; **e**) TGF-β1. ConA (Concanavalin A, 5μg/ml) was used as positive controls, graphs are mean±SEM, N = 10-12 mice /group. **f**) FACS analysis of FoxP3^+^CD4^+^ T cells in thymus (Thy), Mesenteric lymph nodes (Mes) and Spleens (SP), graphs are mean±SEM, N = 4-5 mice / group. FACS analysis of **g**) FoxP3^+^CD4^+^ T_reg_ cells; **i**) FoxA1^+^ T_reg_ cells in CNS infiltrating cells from EAE mice, graphs are mean±SEM, N = 4-5 mice / group.

**Fig. S4** **Transcriptomic profiling of ADAM12-/- T cells revealed dysregulated costimulation, activation & proliferation pathways**

**a**) Functional annotation (based on GO-Term- Biological processes) from David is extracted based on the functional annotation clustering and the top pathways with the greatest number of genes involved in them (p value< 2.50E-02). **b**) Th1 signature genes that show significant (p < 0.05) differences in ADAM12^–/–^ vs ADAM12^+/+^ T cells. **c**) Th17 signature genes expression in ADAM12^–/–^ vs ADAM12^+/+^ T cells.

**Fig. S5** **Adoptive transfer DTH model using MOG_35-55_-reactive T cells vs. sham control T cells.**

Naive CD4 T cells were purified from C57BL/6 spleens, MOG_35-55_-reactive T cells (activated T cells) were prepared from MOG_35-55_ induced EAE mice, then cultured with MOG_35-55_ (50μg/ml) for 3 days. 2x10^6^ MOG_35-55_-reactive T cells or naïve T cells were injected intravenously to SCID mouse tail veins, followed by PBS or MOG_35-55_ injection to mouse ears. **a**) Scheme shows an adoptive transfer model of DTH in immunocompromised SCID mice. 2x10^6^ MOG_35-55_-reactive activated T cells or naive T cells were injected as a sham control group. Then PBS or MOG_35-55_ were injected to mouse ears. **b**) DTH reaction was measured by ear thickness between MOG_35-55_ -injected and PBS-injected after 48 hours. Graphs are mean ± SEM, N = 3 mice per group from one experiment. Student *T* test was used, **p* < 0.05. **c**) H&E staining of ear tissue. Scale bars=100 μm **d**) Histological analysis of ear skin thickness between MOG_35-55_-injected and PBS-injected. Graphs are mean ± SEM, N = 3 mice per group from one experiment. Student *T* test was used, **p* < 0.05.

**Fig. S6 ADAM12 a specific Th1 cell costimulatory molecule important for induction of tissue inflammation**

The schematic drawing is summarizing the findings of this report, in which we show that cell-surface transmembrane ADAM12 acts as a specific costimulatory molecule on a subset of T cells to amplify TCR-signaling, Th1 cell activation and proliferation via induction of the Th1 master transcription factor T-bet and the signature cytokine, IFNγ, immune responses which are prerequisite for induction of neuroinflammation and EAE and skin inflammation in DTH. Under the conditions that T cells lack ADAM12 signaling, this results in defective Th1 cell generation and poor tissue inflammation.
