# Supplementary figures and images for: ADAM12 is a costimulatory molecule that determines Th1 cell fate and mediates tissue inflammation
Source: Cell Mol Immunol. 2020 Jun 22;18(8):1904–19. doi: 10.1038/s41423-020-0486-8 (PMC8322154; doi:10.1038/s41423-020-0486-8)

Supplementary Fig.1

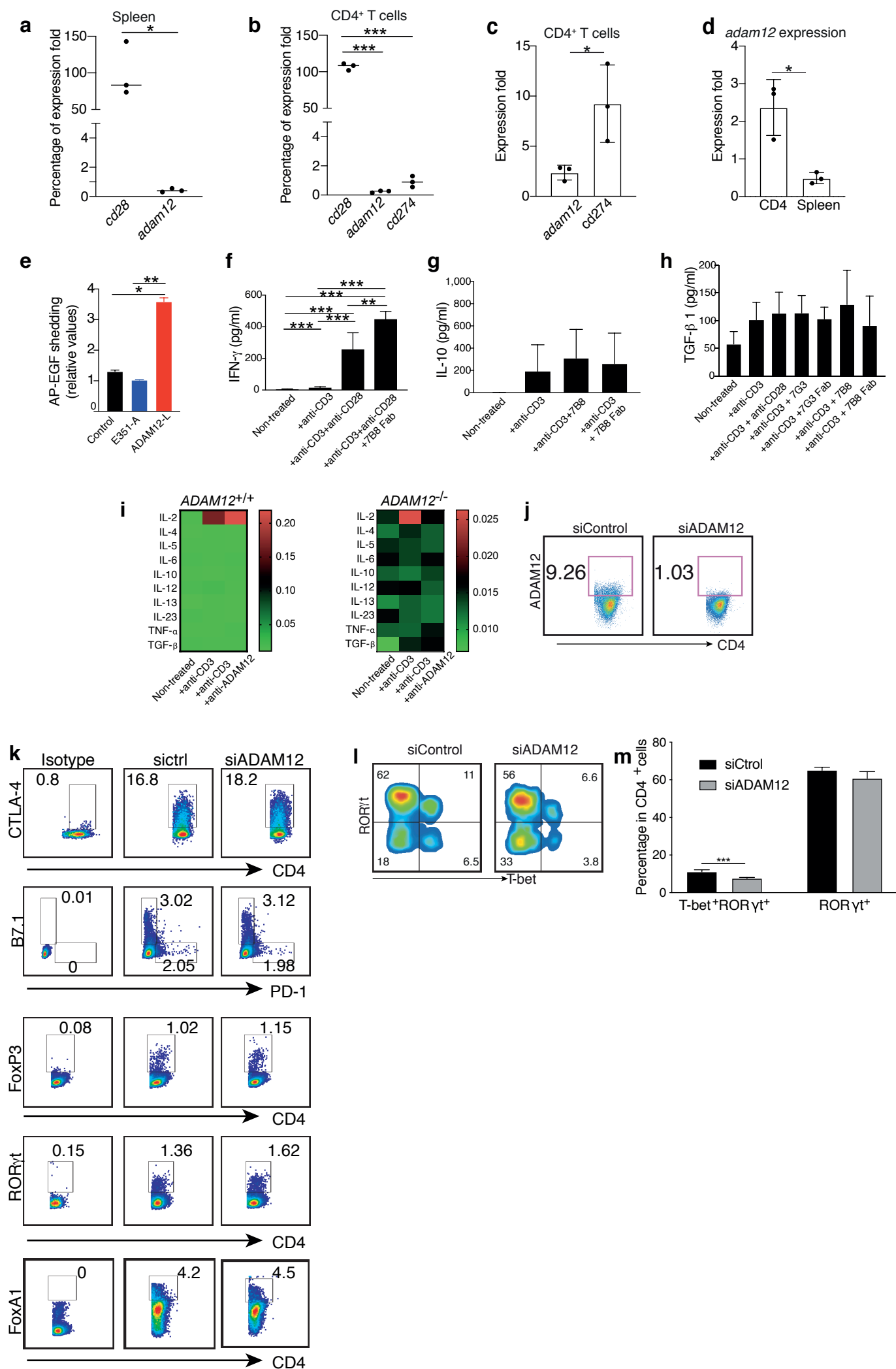

Supplement: Supplementary file 2 — Supplementary Figure 1 [file 41423_2020_486_MOESM2_ESM.pdf]

Supplementary Fig. 2

**a**

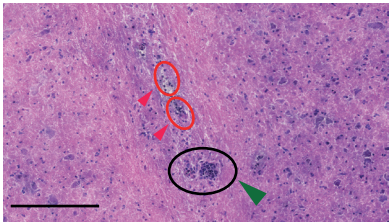

**b**

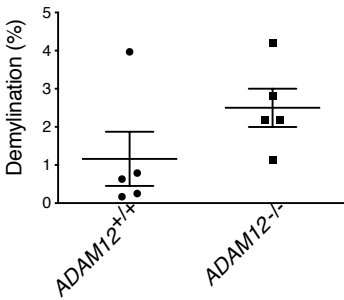

Supplement: Supplementary file 3 — Supplementary Figure 2 [file 41423_2020_486_MOESM3_ESM.pdf]

Supplementary Fig. 3

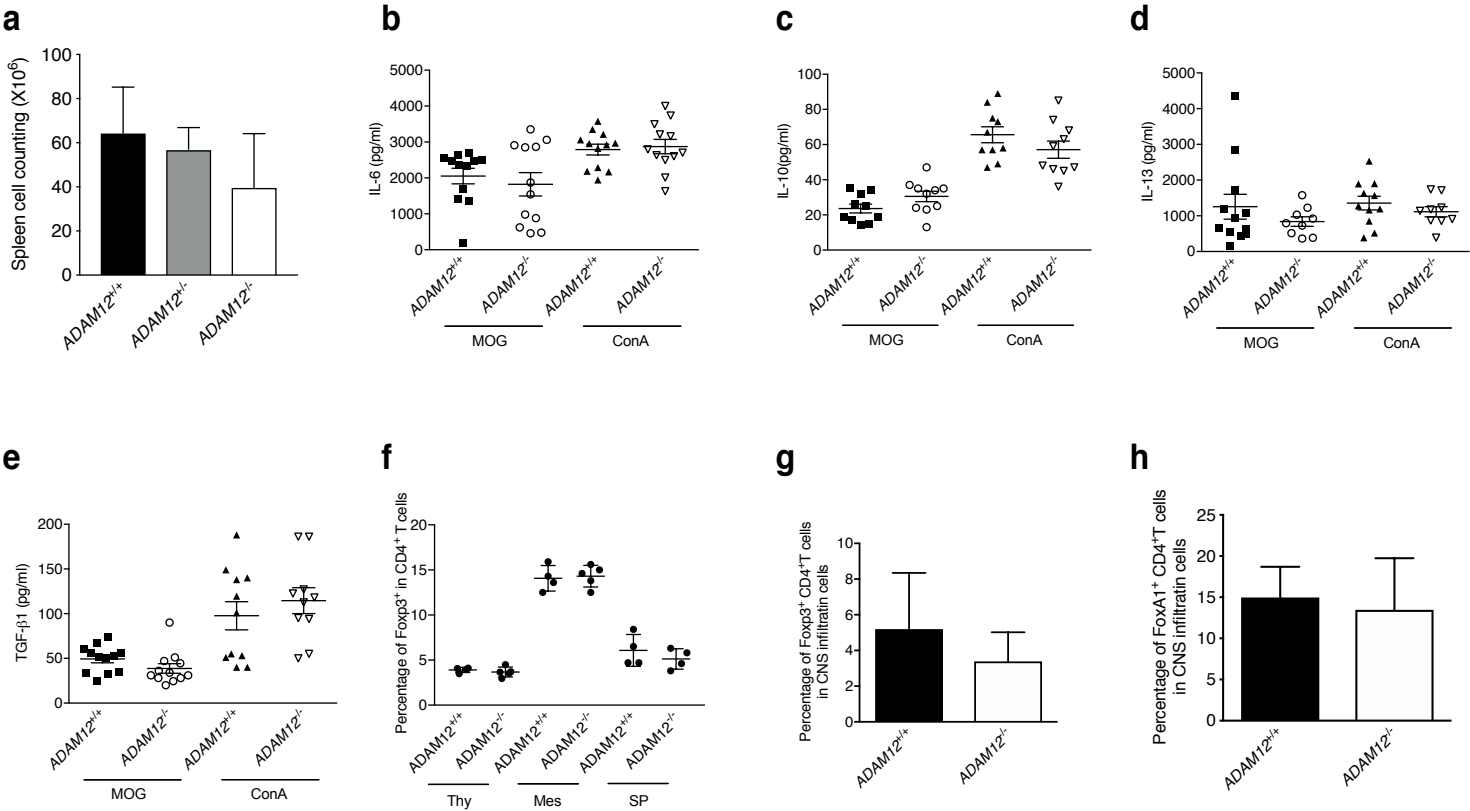

Supplement: Supplementary file 4 — Supplementary Figure 3 [file 41423_2020_486_MOESM4_ESM.pdf]

Supplementary Fig.4

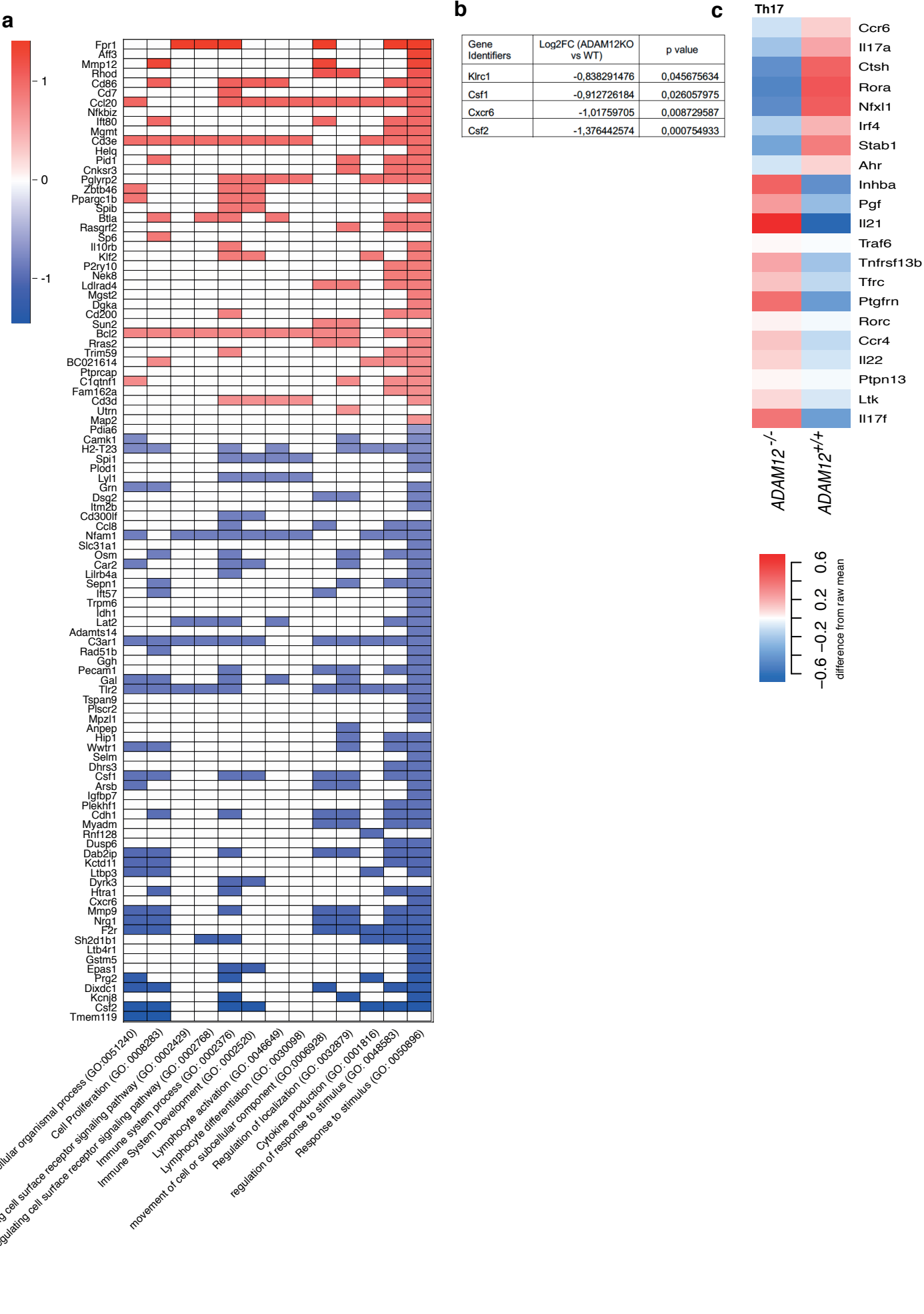

Supplement: Supplementary file 5 — Supplementary Figure 4 [file 41423_2020_486_MOESM5_ESM.pdf]

Supplementary Fig. 5

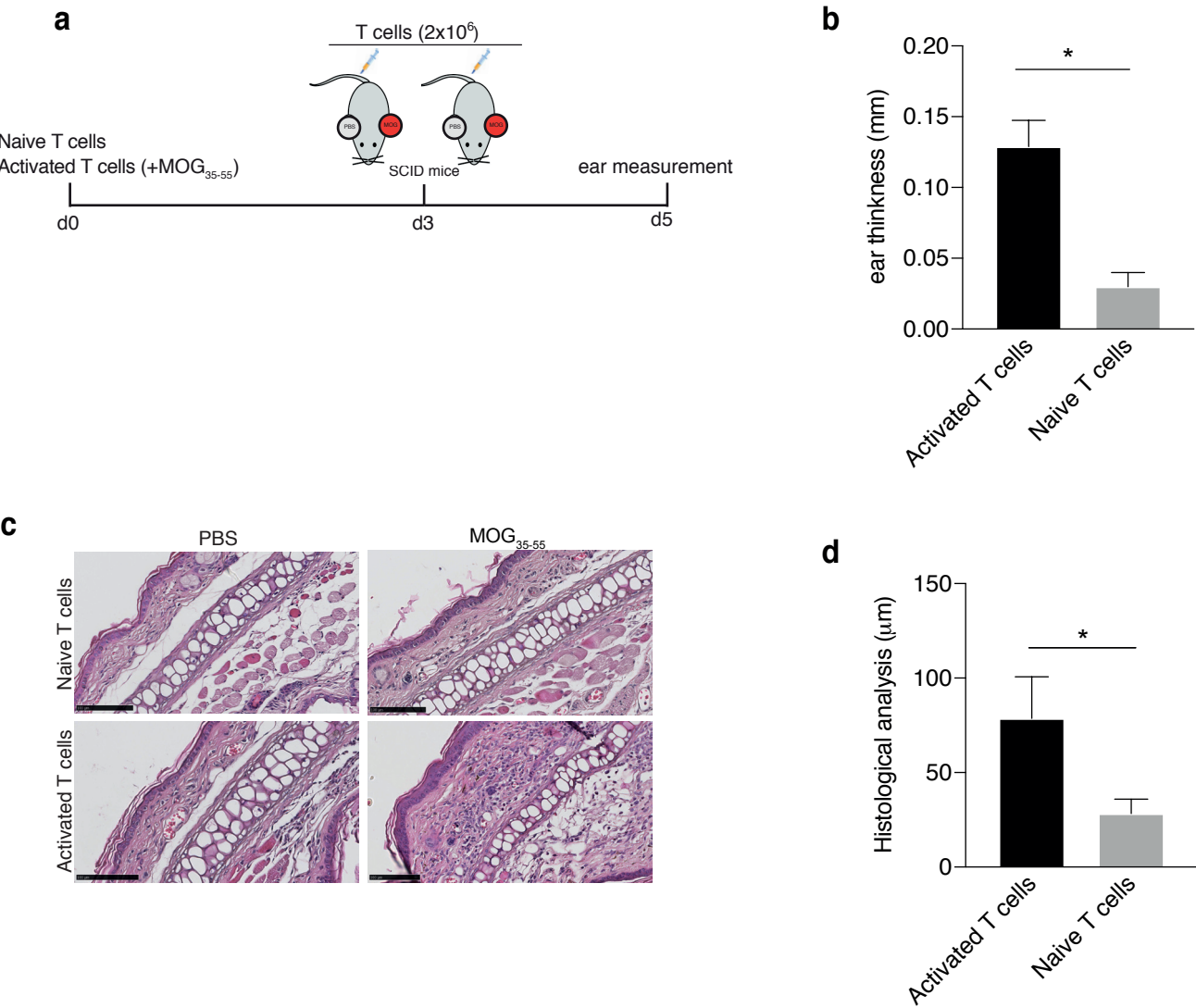

Supplement: Supplementary file 6 — Supplementary Figure 5 [file 41423_2020_486_MOESM6_ESM.pdf]

Supplementary Fig.6

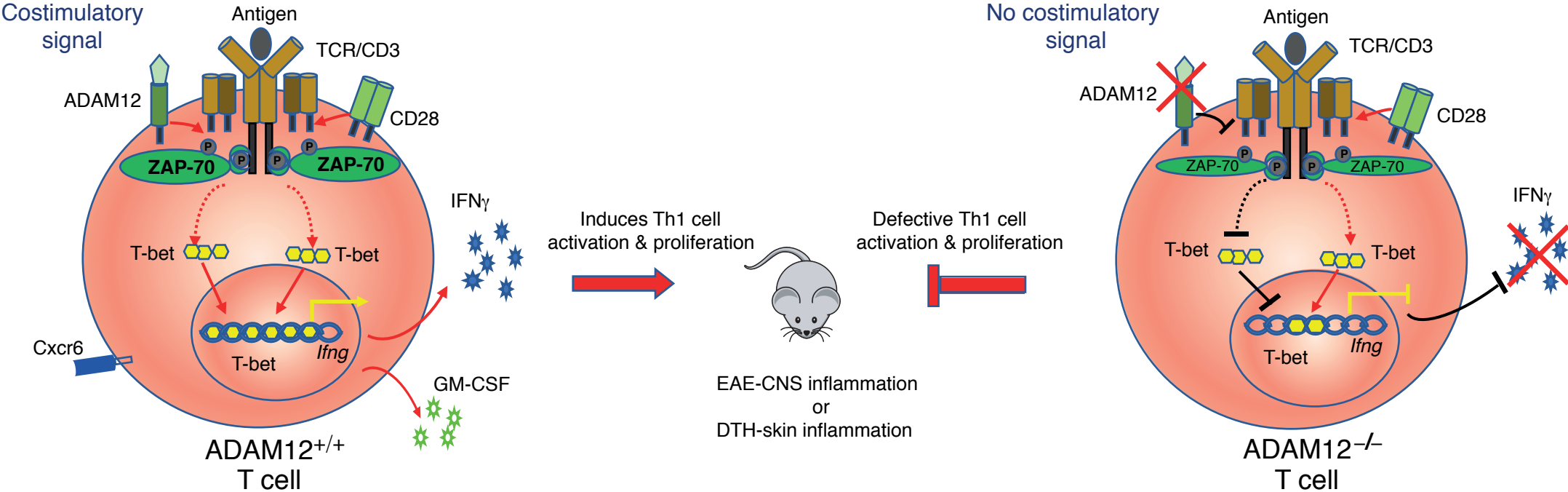

Supplement: Supplementary file 7 — Supplementary Figure 6 [file 41423_2020_486_MOESM7_ESM.pdf]
